# CoCo-ST: Comparing and Contrasting Spatial Transcriptomics data sets using graph contrastive learning

**DOI:** 10.21203/rs.3.rs-4359834/v1

**Published:** 2024-05-20

**Authors:** Muhammad Aminu, Bo Zhu, Natalie Vokes, Hong Chen, Lingzhi Hong, Jianrong Li, Junya Fujimoto, Yuqui Yang, Tao Wang, Bo Wang, Alissa Poteete, Monique B. Nilsson, Xiuning Le, Cascone Tina, David Jaffray, Nick Navin, Lauren A. Byers, Don Gibbons, John Heymach, Ken Chen, Chao Cheng, Jianjun Zhang, Jia Wu

**Affiliations:** 1 Department of Imaging Physics, The University of Texas MD Anderson Cancer Center, Houston, TX, USA.; 2 Department of Thoracic/Head and Neck Medical Oncology, The University of Texas MD Anderson Cancer Center, Houston, TX, USA.; 3 Office of the Chief Technology and Digital Officer, The University of Texas MD Anderson Cancer Center, Houston, TX, USA.; 5 Department of Systems Biology, The University of Texas MD Anderson Cancer Center, Houston, TX, USA.; 6 Department of Bioinformatics and Computational Biology, The University of Texas MD Anderson Cancer Center, Houston, TX, USA.; 7 Institute for Data Science in Oncology, The University of Texas MD Anderson Cancer Center, Houston, TX, USA.; 8 Clinical Research Center, Hiroshima University, Hiroshima, Japan.; 9 Department of Medicine, Institution of Clinical and Translational Research, Baylor College of Medicine, Houston, TX, USA.; 10 These authors contributed equally: Muhammad Aminu, Bo Zhu, Natalie Vokes.; 11 Co-senior authors: Jianjun Zhang, Jia Wu.; 12 Department of Public Health, UT Southwestern Medical Center, Dallas, TX, USA.; 13 Department of Medical Biophysics, University of Toronto, Ontario, Canada.

## Abstract

Traditional feature dimension reduction methods have been widely used to uncover biological patterns or structures within individual spatial transcriptomics data. However, these methods are designed to yield feature representations that emphasize patterns or structures with dominant high variance, such as the normal tissue spatial pattern in a precancer setting. Consequently, they may inadvertently overlook patterns of interest that are potentially masked by these high-variance structures. Herein we present our graph contrastive feature representation method called CoCo-ST (Comparing and Contrasting Spatial Transcriptomics) to overcome this limitation. By incorporating a background data set representing normal tissue, this approach enhances the identification of interesting patterns in a target data set representing precancerous tissue. Simultaneously, it mitigates the influence of dominant common patterns shared by the background and target data sets. This enables discerning biologically relevant features crucial for capturing tissue-specific patterns, a capability we showcased through the analysis of serial mouse precancerous lung tissue samples.

Analyzing spatial transcriptomics (ST) data requires robust feature representation methods to effectively capture the intricate biological information or patterns enriched in these high-dimensional data sets. Although traditional dimension reduction techniques like principal component analysis (PCA^1^) and nonnegative matrix factorization (NMF^2^) have been widely adopted as off-the-shelf approaches for ST data dimension reduction, they primarily aimed at capturing global patterns and variations in the original high-dimensional ST data sets. More recently, the integration of spatial constraints into dimension reduction algorithms has led to the emergence of robust feature representation approaches such as nonnegative spatial factorization^3^, spatial PCA^4^, and MEFISTO^5^. However, these methods tend to prioritize the identification of prominent global patterns with high variability, potentially missing finer localized intrinsic structures marked by lower variability. Furthermore, they are designed to explore one data set at a time and are not tailored to studying the evolutionary dynamics of a tumor microenvironment across multiple data sets. These constraints can result in overlooked information, particularly when studying carcinogenesis, in which tumors progress from a few isolated precancerous sites to invasive cancer across various tissue samples. The majority of these samples exhibit common global patterns (representing normal tissue biology) that may not be of primary interest. Conversely, a small portion of samples contain unique, crucial precancerous structures that require specific attention.

To address these constraints, we proposed a graph contrastive learning framework that we called CoCo-ST (Compare and Contrast Spatial Transcriptomics). CoCo-ST operates by taking two ST data sets as inputs: one serving as the reference (background) and another as the target. These ST data sets typically have certain common structures that are usually not the primary foci. The goal is to extract feature representations that emphasize the new and unique structures enriched in the target ST data set.

In the present study, we used CoCo-ST to thoroughly investigate carcinogenesis using ST data sets from an in-house curated carcinogenesis mouse model. This approach yielded feature representations that enhanced our ability to discern distinctive and noteworthy structures within the target ST data, leading to improvements in downstream analysis.

CoCo-ST was inspired by the recent successes of contrastive learning approaches^6–8^, which learn discriminative feature representations by contrasting positive pairs (similar samples) with negative pairs (dissimilar samples). In our CoCo-ST design workflow (Fig. 1a), we began by collecting tissue samples from mouse lung and processing them using the Visium technology (10x Genomics) to obtain the ST data. We then organized the resulting gene expression data into a gene-spot matrix and further normalized the data to eliminate technical artifacts. CoCo-ST proceeded to construct two weighted graphs, one each for the background and target ST data sets—allowing us to capture the local structures within the data sets. We derived contrastive feature representations by comparing and contrasting the local variances of the background and target graphs. We achieved this by assessing the difference between their respective local total scatter matrices. In the case of a new target ST data set, CoCo-ST simply uses the learned transformation to generate feature representations for the new data (Fig. 1a). These contrastive feature representations can serve as inputs for various other ST analysis tools, for enhanced downstream analysis. We have illustrated the effectiveness of these contrastive feature representations across multiple downstream analysis tasks, including ST data visualization, spatial domain identification, tissue-specific spatial trajectory inference, trajectory inference across multiple tissues, and examination of cell-cell interaction. It is worth mentioning here that CoCo-ST is generically applicable to any ST data types that can be represented in form a gene-spot matrix.

We first applied CoCo-ST to learn transformation by using a mouse normal lung tissue sample (MLP-1) as the background and an abnormal lung tissue sample (MLP-6) containing structures other than the normal spatial domain (Extended Data Fig. 1) as the target. We designated MLP-1 as the background ST data because its spatial structures belong to the normal lung spatial domain, which was also present in all the rest of the tissue samples. We then applied the learned transformation to the remaining tissue samples, resulting in contrastive feature representations that we subsequently used for spatial domain identification (Extended Data Fig. 1) and further downstream analysis. Note, CoCo-ST does not require much data to determine a good transformation compared to the conventional machine learning approaches. Additionally, it has the potential to capture more specific structures within individual samples. These properties make CoCo-ST a valuable complement to large foundation model-based approaches.

Uniform manifold approximation and projection (UMAP) embedding of the learned contrastive features in the target ST data (Extended Data Fig. 2a) illustrated CoCo-ST’s effectiveness in determining feature representations that provide robust discrimination of various spatial structures in the target tissue (Fig. 1b). Clustering the ST data based on the learned contrastive components led to the identification of six clusters, each corresponding to a unique spatial structure. These spatial structures detected using CoCo-ST’s contrastive components agree well with pathologist-annotated regions (Fig. 1b). Spatial clustering of spots based on components determined using the compared Seurat (PCA), STUtility (NMF), NSF and MEFISTO methods failed to effectively detect the hotspot region annotated as hyperplasia by the pathologist (Fig. 1b). Inability to detect spatial structures of low variability affects the performance of the compared methods in detecting the early adenoma (hotspot) region. However, Seurat (PCA) detected the hotspot region but annotated it as belonging to spatial domain 2.

We further annotated the detected spatial structures detected using CoCo-ST based on their differentially expressed marker genes (Extended Data Fig. 2b) and spatial locations. The distribution of these marker genes, including *Epas1* for normal lung tissue (endothelial PAS domain), Slc26a4 for fibrotic/scarred tissue, Cybb for adjacent normal tissue, Hp for the bronchus/alveoli, Ctsh for the adenoma, and Msln for the membrane, showed the expected high expression patterns (Extended Data Fig. 2c). To further validate the adenoma region (hotspot) detected using CoCo-ST, we investigated the most differentially expressed marker genes for the detected adenoma regions and found 3498 marker genes at a false-discovery rate of 5% (Fig. 1c). The most differentially expressed marker genes were domain-specific metagenes for the adenoma region (including the hotspot region). For example, a metagene consisting of *Ctsh*, *Cxcl15*, and *Slc34a2* marked the hotspot region clearly, as these genes exhibited high expression patterns in both the larger adenoma region and smaller hotspot region (Fig. 1e). The *Cxcl15*, and *Slc34a2* genes are uniquely identified by CoCo-ST. The high expression of these genes at both the large and hotspot adenoma regions indicates that these two spatial domains are anatomically similar. Seurat’s inability to identify these important marker genes results to categorizing the hotspot region as belonging to the fibrotic/scarred tissue (Fig. 1b). Also, *Ctsh* gene was reported to be differentially expressed in adenoma region of patients with colorectal cancer^9^. Gene set enrichment analysis of the 10 most differentially expressed marker genes in our study identified biological processes related to lung fibrosis, apoptotic processes, and cell polarity (Extended Data Fig. 2d). For comparison, we also investigated the most differentially expressed marker genes for the compared Seurat (PCA), STUtility (NMF), NSF and MEFISTO methods (Fig. 1d, Extended Data Fig. 2e) based on the learned embedding of these methods and found several genes, most of which marked the larger adenoma region but not the smaller hotspot region. For example, the *Trf* gene was the top marker gene for all of the compared methods (Extended Data Fig. 2e); however, this gene had a high expression pattern in the larger adenoma region but not in the hotspot region (Fig. 1f). These results demonstrated that the compared Seurat (PCA), STUtility (NMF), NSF and MEFISTO methods focus on identifying the main adenoma region with the largest variance, lacking the ability to identify domain-specific metagenes that capture the smaller adenoma structure (hotspot) with relatively low variance.

Examining the weights of the first five contrastive components revealed that CoCo-ST effectively identified major spatial domains (Fig. 2a), indicating that it captured local variations associated with the interesting spatial structures in the target data. For example, component 1 explained variation in multiple spatial domains, which was characterized by large positive weights around the adenoma and alveoli/bronchus and negative weights around the normal lung. Comparing to Seurat (PCA), STUtility (NMF), NSF and MEFISTO, the top components of these methods predominantly focus on the normal lung structure with the largest variance (Fig. 2d). For example, the first components of both Seurat PCA and NSF exhibited larger weights on normal lung structures. Because the first few components of these methods are expected to capture most of the information in the original data and are subsequently used as inputs for downstream analysis, relying solely on these components may result in overlooking crucial biological insights. To gain deeper insight into the underlying biological processes associated with these components, we further investigated the top 20 genes with the largest weights on each of the CoCo-ST’s contrastive components (Fig. 2b). This highlighted individual genes encoding domain-specific signatures such as Retnla, Cyp2f2, Ctsh, Ccl6, and Acta2 (Fig. 2c) as well as gene sets linked with broader biological processes and pathways. Gene set enrichment analysis with the top 20 marker genes for each component revealed enriched gene ontology terms and KEGG pathways specific to each spatial domain. These included heme binding on component 1, retinol metabolism on component 2, IgA immunoglobulin complex on component 3, lysosome on component 4, and extracellular matrix on component 5 (Extended Data Fig. 3).

To investigate the impact of different graph construction methods (molecular vs. spatial) on CoCo-ST’s performance, we constructed a similarity graph based on spatial coordinates rather than gene expression data as done in our prior experiments. This approach has proven highly effective^10^, as it assumes that neighboring spots in the tissue have similar gene expression patterns and likely belong to the same spatial domain. Our findings demonstrated robust CoCo-ST performance when using the similarity graph constructed from the spatial coordinates, effectively identifying the major spatial domains across all target tissue samples (Extended Data Fig. 4). In summary, CoCo-ST demonstrates robust performance with similarity graphs constructed from both spatial coordinates and gene expression data.

Next, we performed deconvolution analysis to infer the cell type composition at each of the spatial domains detected using CoCo-ST. For this analysis, we used matched single-cell RNA sequencing (scRNA-seq) data (Extended Data Fig. 5a) obtained from the same MLP tissue samples as a reference. As expected for the MLP-6 tissue sample (Extended Data Fig. 5b), we observed a concentration of endothelial cells in the normal lung spatial domain (endothelial PAS domain) (Extended Data Figs. 5c,d, 6, 7). The fibrotic/scarred and bronchus/alveoli spatial domains were enriched with fibroblasts. In the adjacent normal spatial domain was an abundance of endothelial cells, whereas the adenoma spatial domain had enrichment of macrophages and proliferating macrophages (Extended Data Figs. 5c,d, 6, 7). Notably, we observed tumor-associated macrophages (TAMs) in the adjacent normal spatial domain (Extended Data Figs. 5c,d, 6, 7), which exhibited significantly upregulated Ccl6. This gene was the top gene with the highest weight on component 4 (Fig. 2c). Of note, component 4 exhibited large weights in spatial regions corresponding to the regions with the highest Ccl6 gene expression. Also, high expression of the Ccl6 gene in a mouse model of lung cancer was reported to be associated with tumor growth and increased metastasis^11^. This evidence underscores the intricate cellular compositions within specific spatial domains, shedding light on potential implications for the progression of lung cancer.

After determining the composition of cell types in the various tissue samples through our deconvolution analysis, we next inferred their communication patterns. Initially, we identified cell-cell interactions by examining ligand-receptor patterns within the individual MLP tissue samples. Our analysis of the MLP-6 tissue sample revealed a strong pattern of communication between endothelial and epithelial cells as well as between endothelial cells and fibroblasts (Extended Data Fig. 5e). Also, we observed strong communication initiating from both proliferating macrophages and B cells within the adenoma spatial domain, indicating an active immune response.

We observed that multiple signaling pathways, including programmed death-ligand 1, GRN, inducible co-stimulator, NECTIN, interleukin-6, WNT, and CXCL, played pivotal roles in cell interactions across different spatial domains. Notably, we predominantly observed WNT ligand-receptor interactions in endothelial cells, epithelial cells, fibroblasts, and macrophages (Extended Data Fig. 5f). Additionally, we observed WNT signaling interactions between proliferating macrophages and B cells, which are enriched in the adenoma spatial domain. Meanwhile, we found self-interaction (among cells of the same group) to be the strongest in proliferating T cells, proliferating macrophages, and endothelial cells (Extended Data Fig. 5g). Network centrality analysis of the inferred WNT signaling network identified TAMs (macrophages and proliferating macrophages) as prominent mediators (gatekeepers) as well as influencers controlling the communication (Extended Data Fig. 5h). Prior studies demonstrated that WNT signaling supports TAMs as drivers of tumor growth and that TAM-derived WNT ligands support tumorigenesis^12^.

We delved deeper into the cell-cell interactions across groups of tissue samples associated with the adenoma and adenocarcinoma spatial domains as determined using CoCo-ST. Specifically, we aggregated the communication weights of multiple tissue samples containing the adenoma (MLP-3, −4, −5, −6, −7, and −9) and adenocarcinoma (MLP-8 and −10) spatial domains to investigate the cell-cell interactions on a broader scale. Of note, we observed a bidirectional interaction between epithelial cells and proliferating macrophages in the adenocarcinoma group (Extended Data Fig. 8a,b) but did not see a similar interaction pattern in the adenoma group (Extended Data Fig. 8c,d). This is consistent with the established role of TAMs in promoting tumor growth and metastasis by engaging in an autocrine loop with cancer cells, thereby stimulating cancer cell progression^13–16^.

Next, we investigated how the normal endothelial, adjacent normal, and tumor spatial domains are connected to each other during tumorigenesis. Specifically, we performed spatial trajectory inference with MLP-6 tissue using the contrastive components derived from CoCo-ST. This analysis revealed a trajectory starting from the normal endothelial domain and moving toward the adjacent normal domain and further into the adenoma spatial domain (Extended Data Fig. 9a,b). To gain a comprehensive view of the trajectory of precancer evolution across the entire population, we combined spots belonging to the adenoma and adenocarcinoma spatial domains as identified by our contrastive components. We then determine a UMAP embedding of the spots (Extended Data Fig. 9c) with which the trajectories were reconstructed (Extended Data Fig. 9d). As seen in Extended Data Fig. 9c, the contrastive components effectively discriminated the three spatial domains and identified a trajectory starting from the normal lung, passing toward the adenoma, and ending at the adenocarcinoma cluster (Extended Data Fig. 9d). These findings align with the well-known biology of mouse tumorigenesis, consisting of a transition from normal tissue to hyperplasia, adenoma, and finally adenocarcinoma. Furthermore, we identified modules of differentially expressed genes that were co-expressed across spots in the different spatial domains as determined using CoCo-ST (Extended Data Fig. 9e,f). Notably, these modules demonstrated high specificity for the different spatial domains, further indicating the effectiveness of CoCo-ST in determining feature representations that captured both the shared and unique spatial structures across the different tissues.

Lastly, we employed CoCo-ST to analyze a publicly available Visium data set generated from mouse brain (anterior and posterior). This data set shows tissue structures that are considerably more complex than the mouse lung precancer data set described above. First, we examined the spatial domain identification performance of CoCo-ST when considering the anterior slice as the reference and the posterior slice as the target and vice versa. The spatial domains detected using CoCo-ST’s contrastive components agree well with the Allen Institute for Brain Science reference atlas diagram (Extended Data Fig. 10a)^17^. We further investigated the top five contrastive components as determined using CoCo-ST for both the anterior and posterior slices. All of these components captured spatial patterns highlighting specific major anatomical regions in the brain (Extended Data Fig. 10b,e). Similar to the mouse precancer model, these components exhibited high component values on specific anatomical regions, such as the cerebral cortex (for anterior component 1) and choroid plexus (for posterior component 2). The top genes for each component (Extended Data Fig. 10c,f) had distinct spatial patterns and exhibited spatial localization to specific brain regions (Extended Data Fig. 10d,g).

To summarize, we introduced an ST feature representation method that opens up the application of graph contrastive learning to ST data analysis. This approach offers significant advantages, particularly in scenarios involving the analysis of multiple ST data sets. It effectively identifies interesting, unique spatial structures in a target ST data set while mitigating the influence of dominant high-variance spatial structures that are common to both target and background ST data sets. Whereas we focused on the ST and Visium platforms, adaptation of CoCo-ST to other platforms such as Xenium, CosMX SMI and MERFISH on which the data can be represented in the form of a gene spot matrix is plausible.

## Methods

### Problem definition and notation

We represented a spatially resolved ST slice from a spatial genomics technology as the set of pairs ${\left\{x_{i},y_{i}\right\}}_{i=1}^{n}$, with $y_{i}\in R^{2}$ denoting a vector of spatial coordinates and $x_{i}\in R^{d}$ denoting a vector of measured gene expression at a corresponding spatial location. We referred to a single spatial location $x_{i}$ as a spot and $s\in \left\{1,2,\ldots ,S_{l}\right\}$ as a slice containing $n_{s}$ spots. Let $X_{s}={\left[x_{1}^{s},x_{2}^{s},\ldots ,x_{n_{s}}^{s}\right]}^{T}$ denote the matrix containing the spot gene expression measurements and $Y_{s}={\left[y_{1}^{s},y_{2}^{s},\ldots ,y_{n_{s}}^{s}\right]}^{T}$ denote the corresponding spatial location matrix from slice s. Worth noting is that the number of spots can differ across different slices and that the slices may be from the same tissue sample or from two different tissue sample.

Our goal is to analyze these $S_{l}$ slices by finding discriminative feature representations that capture the interesting spatial patterns within the different slices. To do this, we identified a background ST data set containing dominant high-variance spatial structures that were present across all slices.

The background ST data play a crucial role in effectively contrasting dominant high-variance spatial structures, which was not the primary focus of this analysis, and in turn assists in detecting the intriguing unique spatial structures enriched in individual target slices. Three key advancements underlie the robust performance of our graph contrastive learning approach. First, we used paired slices to mitigate the impact of spatial structures that are not of primary interest, which subsequently aided the detection of unique spatial structures of particular interest in individual target slices. Second, we constructed local similarity graphs to capture the nuanced local structures in both the background and target ST data sets, thereby ensuring that important spatial structures are not lost. Third, we applied the concept of contrastive learning to compare and contrast the graph embedding of the background and target ST data sets, ensuring that similar spots are positioned close to each other and that dissimilar ones are distanced in the latent space. This collective methodology ensures the accurate identification and representation of distinctive spatial structures.

### Graph representation learning

Recent advances in spatial molecular profiling made graph learning a focus of attention because of the innate resemblance of spatial information to spatial graphs. Graph embedding techniques have great potential for various applications across spatially resolved transcriptomics. Because ST data sets can be represented in a matrix format, we can identify spots as entities of interest and interrogate their interaction. This is equivalent to constructing gene or spot graphs based on suitable similarity measures. Herein we describe the construction of such molecular similarity graphs. An essential task in ST data analysis is to find a lower dimensional manifold space that captures local neighborhood information. Given an ST datum (slice), we can construct a weighted graph G=(V,E) representing complex, non-Euclidean structures, with edges $e_{ij}\in E$ connecting nearby nodes i and j(i,j∈V) to each other if spots $x_{i}^{s}$ and $x_{j}^{s}$ are molecularly similar. A natural variation of this graph is to construct a graph of k-nearest neighbors in which similarity of nodes is usually quantified using the Euclidean metric (i.e., nodes i and j are connected by an edge $e_{ij}$ if $x_{i}^{s}$ is among the k-nearest neighbors of $x_{j}^{s}$ or $x_{j}^{s}$ is among the k-nearest neighbors of $x_{i}^{s}$). The graph structure G=(V,E) is commonly encoded in an $n_{S}\times n_{s}$ affinity matrix S with entries in [0, 1] and takes large values if $x_{i}^{s}$ and $x_{j}^{s}$ are close (or similar). Several approaches to computing the affinity matrix S are available, one of which is the heat kernel weighting technique depicted by the equation

(1) $$S_{ij}^{s}=\left\{\begin{array}{ll} e^{-\frac{{∥x_{i}^{s}-x_{j}^{s}∥}^{2}}{t}}, & \text{if}\;x_{i}^{s}\in N\left(x_{j}^{s}\right)\text{or}\;x_{j}^{s}\in N\left(x_{i}^{s}\right),\\ 0, & \;\;\;\;\;\text{Otherwise} \end{array}\right.$$

where $N\left(x_{j}^{s}\right)$ denotes the set of k-nearest neighbors of $x_{j}^{s}$ and t is a user-specified parameter.

Based on the graph construction approach described above, the similarity among spots is quantified based on gene expression measurements at the corresponding spots. However, because gene expression measurements are captured alongside its spatial information in ST, these spatial locations can be used to construct similarity graphs. The spatial graphs constructed in this way are similar to molecular similarity graphs in the sense that nodes correspond to spots. However, edges capture proximity of spots in the $R^{2}$ coordinate space. The affinity matrix with the spatial locations can now be constructed as

(2) $$S_{ij}^{s}=\left\{\begin{array}{ll} e^{-\frac{{∥y_{i}^{s}-y_{j}^{s}∥}^{2}}{t}}, & \text{if}\;y_{i}^{s}\in N\left(y_{j}^{s}\right)\text{or}\;x_{j}^{s}\in N\left(x_{i}^{s}\right).\\ 0, & \;\;\;\;\text{Otherwise} \end{array}\right.$$

Also, the spatial graph can be constructed using both the spatial locations and the molecular profiles treated as node features. Graph representation learning approaches are considered to determine biologically meaningful representations of these graphs by finding meaningful lower dimensional representations of nodes present in a complex graph, where local structures in the data are well captured. A widely used criterion for determining such a representation is to solve the objective function

(3) $$\underset{W}{\text{min}}\sum_{i,j=1}^{n_{s}}{\left\|z_{i}^{s}-z_{j}^{s}\right\|}^{2}S_{ij}^{s},$$

where $z_{i}^{s}=W^{T}x_{i}^{s}$ denotes the lower dimensional representation of $x_{i}^{s}$. Solving Eq. (3) under appropriate constraints ensures that if $x_{i}^{s}$ and $x_{j}^{s}$ are similar (or nodes i and j are connected in the graph), then $z_{i}^{s}$ and $z_{j}^{s}$ are similar (close), as well.

### Contrastive representation learning

Contrastive learning has recently emerged as a successful method of unsupervised graph representation learning. Contrastive learning methods first perform augmentation of the input data and enforce via a suitable objective function mapping of augmentation of the same data (positive pairs) close to each other in the representation (latent) space and augmentation of different data (negative pairs) far apart from each other. Arguably, a low-dimensional representation that is near optimal in the contrastive objective function is guaranteed to linearly separate similar data from dissimilar data. Such representations provide competitive performance in a host of downstream tasks. In early visual representation learning studies, researchers leveraged a pixel as local view to conduct local-to-local^18^ or local-to-global^19^ contrastive learning, whereas researchers recently found that randomly cropped image snippets help contrastive models better capture the relationships between image elements^6^. This motivated us to perform contrastive representation learning at the global image level.

Like several other machine learning approaches, contrastive representation learning can be performed in an unsupervised (self-supervised) or supervised learning strategy. In self-supervised settings, contrastive learning methods learn discriminative feature representations based on some similarity measure defined according to the data. Consider the objective function defined by^20^

(4) $$L_{1}=(1-Y)*\frac{1}{2}{∥x_{i}-x_{j}∥}^{2}+\frac{Y}{2}*{\left\{\text{max}\left(0,m-{∥x_{i}-x_{j}∥}^{2}\right)\right\}}^{2},$$

where m>0 is a hyperparameter defining the lower bound distance between dissimilar samples, Y is a binary label with Y=0 if $x_{i}$ and $x_{j}$ are similar, and Y=1 if $x_{i}$ and $x_{j}$ are dissimilar. Minimizing the objective function is an attempt to determine a lower dimensional manifold subspace where similar input samples are mapped nearby and dissimilar samples are far apart. When sample labels are available, they can be integrated into the definition of similarity and dissimilarity to better guide the contrastive model to mapped samples belonging to the same class (same label) close to each other and samples of different classes farther apart. This approach is referred to as supervised contrastive representation learning. Both the self-supervised and fully supervised contrastive learning approaches are powerful methods of learning discriminative feature representations.

### Graph contrastive feature representation using CoCo-ST

Most of the traditional feature representation approaches are designed to determine feature representations through maximization of data variance. These approaches can perform poorly if the ST data structures with maximal variances are not the structures of interest, as the local structures of interest are masked by the dominant high-variance structures. The feature representations determined using these approaches capture little to no useful information reflecting the unique low-variance local structures present in the ST data, which are usually treated as noise. Also, these traditional approaches are designed to explore one ST data set at a time, which can hinder their performance in cases where there are multiple interconnected data sets that need to be explored.

To overcome these limitations, we propose CoCo-ST, which compares and contrasts the global and local variances in ST data sets to better capture discriminant and structural information. More generally, we use two ST data sets (background and target) and subsequently construct two similarity graph views: one for the background ST data set and the other for the target ST data set. We then design a contrastive objective function to learn feature representations that capture high global (and/or local) variances enriched in the target ST data while simultaneously attaining small global (and/or local) variances in the background ST data. Given a background ST data set $X_{b}={\left[x_{1}^{b},x_{2}^{b},\ldots ,x_{n_{b}}^{b}\right]}^{T}$ containing spatial structures of no primary interest, such as a normal lung region, we can use the following two terms to measure the smoothness of the lower dimensional representation:

(5) $${ℛ}_{1}=\underset{W}{\text{min}}\sum_{i=1}^{n_{b}}{∥x_{i}^{b}-WW^{T}x_{i}^{b}∥}^{2}\;=\underset{W}{\text{max}}tr\left(W^{T}X_{b}X_{b}^{T}W\right)$$

and

(6) $${ℛ}_{2}=\underset{W}{\text{min}}\overset{n_{b}}{\underset{i,j=1}{\sum }}{∥W^{T}x_{i}^{b}-W^{T}x_{j}^{b}∥}^{2}S_{ij}^{b}\;=\underset{W}{\text{min}}\left(\overset{n_{b}}{\underset{i,j=1}{\sum }}W^{T}x_{i}^{b}D_{ii}^{b}{\left(x_{i}^{b}\right)}^{T}W^{T}-W^{T}x_{i}^{b}S_{ij}^{b}{\left(x_{j}^{b}\right)}^{T}W^{T}\right)\;=\underset{W}{\text{min}}\;\text{tr}\left(W^{T}X_{b}L^{b}X_{b}^{T}W\right),$$

where tr(⋅) is the trace operator, D is a diagonal matrix whose entries are the column (or row) sums of $S,D_{ii}=\sum_{k}S_{ik}$, and L=D-S is the graph Laplacian matrix. We consider the symmetric normalized graph Laplacian matrix $\overset{‾}{L}=D^{-1/2}LD^{-1/2}$ in our later derivations. By minimizing ${ℛ}_{1}$, we aim to minimize the reconstruction error, whereas minimizing ${ℛ}_{2}$ is an attempt to preserve the local structure (i.e., if two spots $x_{i}^{s}$ and $x_{j}^{s}$ are molecularly similar, their low-dimensional representations $W^{T}x_{i}^{b}$ and $W^{T}x_{j}^{b}$ are also similar). Combining Eqs. (5) and (6), we can have the equivalent formulation

(7) $${𝒪}_{1}=\underset{W^{T}W=I}{\text{max}}tr\left(W^{T}X_{b}X_{b}^{T}W\right)-\mu_{1}tr\left(W^{T}X_{b}{\overset{‾}{L}}^{b}X_{b}^{T}W\right)\;=\underset{W^{T}W=I}{\text{max}}tr\left(W^{T}X_{b}H_{b}X_{b}^{T}W\right),$$

where $H_{b}=I-\mu_{1}{\overset{‾}{L}}^{b},I$ is an identity matrix, ${\overset{‾}{L}}^{b}$ is the normalized graph Laplacian for the background ST data, and $0\leq \mu_{1}\leq 1$ is a hyperparameter that controls the smoothness of the new representation. The matrix $H_{b}=I-\mu_{1}{\overset{‾}{L}}^{b}$ can be considered a graph Laplacian filter^21^ that helps smooth the data while preserving underlying spatial structures in an ST slice.

Similarly, for a target ST data set $X_{t}={\left[x_{1}^{t},x_{2}^{t},\ldots ,x_{n_{t}}^{t}\right]}^{T}$ containing unique, interesting spatial structures, we can write the formulation

(8) $${𝒪}_{2}=\underset{W^{T}W=I}{\text{max}}tr\left(W^{T}X_{t}H_{t}X_{t}^{T}W\right),$$

where $H_{t}=I-\mu_{2}{\overset{‾}{L}}^{t},{\overset{‾}{L}}^{t}$ is the normalized graph Laplacian for the target ST data and $0\leq \mu_{2}\leq 1$ is a hyperparameter.

Combining Eqs. (7) and (8), CoCo-ST solves the following objective function

(9) $${𝒪}_{3}=\underset{W^{T}W=I}{\text{max}}tr\left(W^{T}X_{t}H_{t}X_{t}^{T}W\right)-\eta tr\left(W^{T}X_{b}H_{b}X_{b}^{T}W\right),$$

where η≥0 is the contrastive parameter that determines the tradeoff between high target global (and/or local) variance and low background global (and/or local) variance. We will first describe how to maximize the objective function ${𝒪}_{3}$. Let Λ be the Lagrange multiplier for the constraint $W^{T}W=I$. The Lagrange ℒ is

(10) $$ℒ=tr\left(W^{T}X_{t}H_{t}X_{t}^{T}W\right)-\eta tr\left(W^{T}X_{b}H_{b}X_{b}^{T}W\right)-\Lambda tr\left(W^{T}W-I\right).$$

The partial derivative of ℒ with respect to W is

(11) $$\frac{\partial ℒ}{\partial W}=X_{t}H_{t}X_{t}^{T}W-\eta X_{b}H_{b}X_{b}^{T}W-\Lambda \text{W}.$$

The optimum solution to Eq. (10) satisfies $\frac{\partial ℒ}{\partial W}=0$. We therefore have

(12) $$X_{t}H_{t}X_{t}^{T}W-\eta X_{b}H_{b}X_{b}^{T}W-\Lambda \text{W}=0\;\left(X_{t}H_{t}X_{t}^{T}-\eta X_{b}H_{b}X_{b}^{T}\right)W=\Lambda \text{W}.$$

Thus, the transformation matrix that maximizes the objective function ${𝒪}_{3}$ can be obtained by solving the eigenvalue problem (Eq. 12). Let $w_{1},w_{2},\ldots ,w_{p}$ be the eigenvectors from Eq. (12) corresponding to the top p largest eigenvalues $\lambda_{1}\geq \lambda_{2}\geq \cdots ,\geq \lambda_{p}$. The lower dimensional manifold representation can then be obtained as follows:

(13) $$x_{i}^{t}\to z_{i}^{t}=W^{T}x_{i}^{t}\;W^{T}=\left[w_{1},w_{2},\ldots ,w_{p}\right],$$

where $z_{i}^{t}$ is a p-dimensional representation of $x_{i}^{t}$, and W is a d×p matrix. This feature representation preserves the local structure of the ST data sets. A step-by-step description of the proposed CoCo-ST method is summarized in Algorithm 1.

**Algorithm 1.** CoCo-ST.

Input: Background $X_{b}={\left[x_{1}^{b},x_{2}^{b},\ldots ,x_{n_{b}}^{b}\right]}^{T}$ and target $X_{t}={\left[x_{1}^{t},x_{2}^{t},\ldots ,x_{n_{t}}^{t}\right]}^{T}$ ST data sets, together with corresponding spatial locations $Y_{b}={\left[y_{1}^{b},y_{2}^{b},\ldots ,y_{n_{b}}^{b}\right]}^{T}$ and $Y_{t}={\left[y_{1}^{t},y_{2}^{t},\ldots ,y_{n_{t}}^{t}\right]}^{T}$, the number of nearest neighbors (k), and the hyperparameters $\mu_{1},\mu_{2}$ and η.
Output: The low-dimensional contrastive feature representations for the target ST data $Z_{t}=W^{T}X_{t}$
Construct the adjacency matrix for both the background and target ST data sets according to Eq. (1) or (2). Construct the normalized graph Laplacian matrices ${\overset{-}{L}}^{b}$ and ${\overset{-}{L}}^{t}$ together with the graph Laplacian filters $H_{b}=I-\mu_{1}{\overset{-}{L}}^{b}$ and $H_{t}=I-\mu_{2}{\overset{-}{L}}^{t}$. Compute the matrices $X_{b}H_{b}X_{b}^{T}$ and $X_{t}H_{t}X_{t}^{T}$. Solve the eigenvalue problem in Eq. (12). Compute the low-dimensional contrastive feature representations for the target ST data as $Z_{t}=W^{T}X_{t}$.

We next investigate the computational complexity of the proposed CoCo-ST algorithm. Its complexity is dominated mainly by three parts: local similarity graph construction, matrix multiplication, and solving an eigenvalue problem. Assuming we have $n_{b}$ and $n_{t}$ spots in d-dimensional spaces (d gene expression measurements) for the background and target ST data sets, to construct the similarity graphs, we first perform a k-nearest neighbor search for both data sets. The distance between any two spots in the background ST data can be computed in $O\left(dn_{b}^{2}\right)$, and the k-nearest neighbors can be found with $O\left(kn_{b}^{2}\right)$. Thus, the k-nearest neighbor search for the background and target ST data sets has complexities $O\left((d+k)n_{b}^{2}\right)$ and $O\left((d+k)n_{t}^{2}\right)$, respectively. The complexities for computing the matrices $X_{b}H_{b}X_{b}^{T}$ and $X_{t}H_{t}X_{t}^{T}$ are $O\left(\left(n_{b}^{2}+n_{b}d\right)d\right)$ and $O\left(\left(n_{t}^{2}+n_{t}d\right)d\right)$, respectively. The last part is computing the eigenvectors corresponding to the top p eigenvalues of the eigenproblem in Eq. (12), whose complexity is $O\left(pd^{2}\right)$. Therefore, the time complexity of the CoCo-ST algorithm is $O\left((d+k)\left(n_{b}^{2}+n_{t}^{2}\right)+\right.\left.\left(\left(n_{b}+d\right)n_{b}+\left(n_{t}+d\right)n_{t}+pd\right)d\right)$. Because $k\ll n_{b}$ (or $n_{t}$) and p≪d, the overall complexity of CoCo-ST is determined by the number of spots $n_{b}$ (or $n_{t}$) and the number of genes (d).

Several aspects of the proposed CoCo-ST approach are worth highlighting. Specifically:

1. If $\mu_{1}=\mu_{2}=0$, the matrices $X_{t}H_{t}X_{t}^{T}$ and $X_{b}H_{b}X_{b}^{T}$ reduce to $X_{t}X_{t}^{T}$ and $X_{b}X_{b}^{T}$, respectively, so the objective function $\left({𝒪}_{3}\right)$ reduces to that of contrastive PCA (cPCA)^22^. Therefore, cPCA can be regarded as a variant of CoCo-ST.
2. Whereas cPCA and the majority of the traditional feature representation approaches focus on global geometrical structures, CoCo-ST can exploit the intrinsic geometric structures of ST data sets and incorporate them as additional regularization terms. Through construction of a graph to model local geometric structures, CoCo-ST can have more discriminating power than cPCA and the traditional feature representation approaches.
3. CoCo-ST simultaneously learns both global and local-level representations to complement tissue-wide representations, enabling it to distinguish different spatial areas in an ST tissue slice.
4. The graphs in our proposed CoCo-ST approach are solely unsupervised and constructed from molecular data or spatial location information. Other information, such as label information, can also be used to guide graph construction, leading to other extensions of CoCo-ST such as supervised or semisupervised CoCo-ST.
5. The proposed CoCo-ST approach differs from existing graph contrastive learning approaches that focus on graph neural network architectures for graph structured data. CoCo-ST considers the gene expression data and tries to learn local representations to better capture ST data structural information. As such, the objective functions of CoCo-ST and the conventional graph neural networks are different.

### Why is CoCo-ST good for ST data analysis?

CoCo-ST imposes molecularly or spatially similar spots to have similar feature representations, by which the intrinsic geometric structure of the ST data tends to be preserved. This is a useful property in ST data analysis because interesting spatial structures will not be lost owing to feature representation. In addition, CoCo-ST determines its discriminant (contrastive) feature representations from both the background and target ST data sets and thus can provide even more discriminative feature representations than the traditional approaches that focus only on a single ST data set. To explain this, we provided the following remarks and theorem.

### Remark 1

When η=0, CoCo-ST degenerates to a feature representation method that determines its discriminant vectors from the range space of the matrix $X_{t}H_{t}X_{t}^{T}$ associated with the target data alone. When η>0, the matrix $X_{t}H_{t}X_{t}^{T}-\eta X_{b}H_{b}X_{b}^{T}$ is not guaranteed to be positive semidefinite even though $X_{t}H_{t}X_{t}^{T}$ and $X_{b}H_{b}X_{b}^{T}$ are both symmetric and positive semidefinite. Let w be the eigenvector of the matrix $X_{t}H_{t}X_{t}^{T}-\eta X_{b}H_{b}X_{b}^{T}$ corresponding to the eigenvalue λ<0. We then have

$$\left(X_{t}H_{t}X_{t}^{T}-\eta X_{b}H_{b}X_{b}^{T}\right)w=\lambda w$$

$$wX_{t}H_{t}X_{t}^{T}w=\eta wX_{b}H_{b}X_{b}^{T}w+\lambda$$

$$\frac{wX_{t}H_{t}X_{t}^{T}w}{wX_{b}H_{b}X_{b}^{T}w}=\eta +\frac{\lambda }{wX_{b}H_{b}X_{b}^{T}w}$$

Because both $X_{t}H_{t}X_{t}^{T}$ and $X_{b}H_{b}X_{b}^{T}$ are positive semidefinite, we can conclude that

$$\frac{wX_{t}H_{t}X_{t}^{T}w}{wX_{b}H_{b}X_{b}^{T}w}=\eta +\frac{\lambda }{wX_{b}H_{b}X_{b}^{T}w}\geq 0$$

Thus, the eigenvectors corresponding to the negative eigenvalues are derived from the range space of $X_{b}H_{b}X_{b}^{T}$ and contain some discriminant information.

### Theorem 1

Suppose the matrix $X_{b}H_{b}X_{b}^{T}$ is singular and that w is an eigenvector of the matrix $X_{t}H_{t}X_{t}^{T}-\eta X_{b}H_{b}X_{b}^{T}$ corresponding to the eigenvalue λ>0. The eigenvector w is then in the null space of $X_{b}H_{b}X_{b}^{T}$ when η→∞.

**Proof**. Because w is the eigenvector of the matrix $X_{t}H_{t}X_{t}^{T}-\eta X_{b}H_{b}X_{b}^{T}$ corresponding to the eigenvalue λ>0, we have

$$\left(X_{t}H_{t}X_{t}^{T}-\eta X_{b}H_{b}X_{b}^{T}\right)w=\lambda w$$

$$wX_{b}H_{b}X_{b}^{T}w=\frac{1}{\eta }\left(wX_{t}H_{t}X_{t}^{T}w-\lambda \right)$$

Since λ>0, we have the following:

$$wX_{b}H_{b}X_{b}^{T}w<\frac{1}{\eta }wX_{t}H_{t}X_{t}^{T}w$$

Of note is that both $X_{t}H_{t}X_{t}^{T}$ and $X_{b}H_{b}X_{b}^{T}$ are positive semidefinite (i.e., $wX_{t}H_{t}X_{t}^{T}w\geq 0$ and $wX_{b}H_{b}X_{b}^{T}w\geq 0$). As a result, we have

$$\underset{\eta \to \infty }{\text{lim}}wX_{b}H_{b}X_{b}^{T}w=0$$

■

Thus, as η→∞, the eigenvectors corresponding to the positive eigenvalues belong to the null space of $X_{b}H_{b}X_{b}^{T}$.

### Remark 2

As η→∞, the eigenvectors corresponding to the positive eigenvalues of the eigenproblem (Eq. [12]) contain the most discriminant information. We can rewrite the eigenvalue problem (Eq. [12]) as

$$\left(X_{t}H_{t}X_{t}^{T}-\eta X_{b}H_{b}X_{b}^{T}\right)w=\lambda w$$

$$wX_{t}H_{t}X_{t}^{T}w=\eta wX_{b}H_{b}X_{b}^{T}w+\lambda$$

$$\frac{wX_{t}H_{t}X_{t}^{T}w}{wX_{b}H_{b}X_{b}^{T}w}\to \infty$$

Thus, as η→∞, the eigenvectors corresponding to the positive eigenvalues contain the most discriminant information.

### Remark 3

As η→∞, the eigenvectors corresponding to the zero eigenvalues of the eigenproblem (Eq. [12]) contain no discriminant information. When λ=0, the eigenvalue problem reduces to

$$\left(X_{t}H_{t}X_{t}^{T}-\eta X_{b}H_{b}X_{b}^{T}\right)w=\lambda w=0$$

$$wX_{t}H_{t}X_{t}^{T}w=\eta wX_{b}H_{b}X_{b}^{T}w$$

Since $wX_{t}H_{t}X_{t}^{T}w$ and $wX_{b}H_{b}X_{b}^{T}w$ are finite and η→∞, we have

$$wX_{t}H_{t}X_{t}^{T}w=0,wX_{b}H_{b}X_{b}^{T}w=0$$

Thus, the eigenvectors corresponding to the zero eigenvalues contain no discriminant information, as η→∞. In general, we can conclude that CoCo-ST derives its discriminant feature vectors from the range spaces of both $X_{t}H_{t}X_{t}^{T}$ and $X_{b}H_{b}X_{b}^{T}$. The parameter η can be used to balance the contribution from the two spaces. Moreover, by extracting the eigenvectors of the eigenvalue problem in Eq. (12) corresponding to the largest positive eigenvalues, CoCo-ST can capture the most discriminant information in both the background and target ST data sets, enabling effective identification of the interesting spatial structures enriched in the target ST data set.

### Nonlinear extension of CoCo-ST

Thus far, we have focused on linear feature representation. However, biological data are well known to be complex and highly nonlinear. Therefore, we extended CoCo-ST to perform nonlinear feature representation in a reproducing kernel Hilbert space ℋ, which gives rise to nonlinear CoCo-ST. We considered nonlinear mapping ϕ(⋅) of both the background $X_{b}$ and target $X_{t}$ ST data sets from the original input spaces to ℋ. Let $\Phi_{b}$ and $\Phi_{t}$ denote the background and target ST data sets in ℋ:

$$\Phi_{b}={\left[\phi \left(x_{1}^{b}\right),\phi \left(x_{2}^{b}\right),\ldots ,\phi \left(x_{n_{b}}^{b}\right)\right]}^{T}$$

$$\Phi_{t}={\left[\phi \left(x_{1}^{t}\right),\phi \left(x_{2}^{t}\right),\ldots ,\phi \left(x_{n_{t}}^{t}\right)\right]}^{T}$$

Denote by V the projection matrix in ℋ. The corresponding objective function $\left({𝒪}_{3}\right)$ of CoCo-ST in ℋ is

(14) $${𝒪}_{4}=\underset{V^{T}V=I}{\text{max}}tr\left(V^{T}\Phi_{t}H_{t}\Phi_{t}^{T}V\right)-\eta tr\left(V^{T}\Phi_{b}H_{b}\Phi_{b}^{T}V\right).$$

Let $N=n_{b}+n_{t}$, and define the data $q_{1},q_{2},\ldots ,q_{N}$ by

$$q_{i}=\left\{\begin{array}{ll} x_{i}^{t}, & \text{if}\;1\leq i\leq n_{t}\\ x_{i-n_{t^{\prime }}}^{b} & \;\;\text{otherwise} \end{array}\right.$$

Since the projection vectors $v_{1},v_{2},\ldots ,v_{p}$ (column vectors in V) are linear combinations of $\phi \left(q_{1}\right),\phi \left(q_{2}\right),\ldots ,\phi \left(q_{N}\right)$, coefficients $\alpha_{i},i=1,2,\ldots ,N$ exist such that

$$v_{k}=\sum_{i=1}^{N}\alpha_{i}\phi \left(q_{i}\right)=\Phi_{c}\alpha \;\Rightarrow V=\Phi_{c}\;\text{A}$$

where $\alpha ={\left(\alpha_{1},\alpha_{2},\ldots ,\alpha_{N}\right)}^{T}\in R^{N},\text{A}=\left[\alpha^{1},\alpha^{2},\ldots ,\alpha^{p}\right]$. Following some algebraic formulations, we can rewrite the objective function $\left({𝒪}_{4}\right)$ in the following equivalent form:

(15) $${𝒪}_{4}=\underset{{\text{A}}^{T}\Phi_{c}^{T}\Phi_{c}\text{A}=1}{\text{max}}tr\left({\text{A}}^{T}\Phi_{c}^{T}\Phi_{t}H_{t}\Phi_{t}^{T}\Phi_{c}\text{A}\right)-\eta tr\left({\text{A}}^{T}\Phi_{c}^{T}\Phi_{b}H_{t}\Phi_{b}^{T}\Phi_{c}\text{A}\right)\;=\underset{{\text{A}}^{T}K_{cc}\text{A}=1}{\text{max}}tr\left({\text{A}}^{T}K_{ct}H_{t}K_{tc}\text{A}\right)-\eta tr\left({\text{A}}^{T}K_{cb}H_{b}K_{bc}\text{A}\right),$$

where $K_{cc}=\Phi_{c}^{T}\Phi_{c},K_{ct}=\Phi_{c}^{T}\Phi_{t},K_{tc}=\Phi_{t}^{T}\Phi_{c},K_{cb}=\Phi_{c}^{T}\Phi_{b}$, and $K_{bc}=\Phi_{b}^{T}\Phi_{c}$ are the kernel matrices. Several choices of the kernel functions are available, including the polynomial kernel $\text{κ}\left(x_{i}^{t},x_{i}^{b}\right)={\left({\left(x_{i}^{t}\right)}^{T}x_{i}^{b}+1\right)}^{d}$; Gaussian kernel $\text{κ}\left(x_{i}^{t},x_{i}^{b}\right)=\text{exp}\left(-\frac{{∥x_{i}^{t}-x_{i}^{b}∥}^{2}}{\sigma^{2}}\right)$; and sigmoid kernel $\text{κ}\left(x_{i}^{t},x_{i}^{b}\right)=\left({\left(x_{i}^{t}\right)}^{T}x_{i}^{b}+\gamma \right)$.

Following approach similar to that in linear CoCo-ST, the projection vectors in Eq. (15) can be obtained as the eigenvectors corresponding to the top p largest eigenvalues of the generalized eigenvalue problem

(16) $$\left(K_{ct}H_{t}K_{tc}-\eta K_{cb}H_{b}K_{bc}\right)\text{A}=\Lambda K_{cc}\text{A}$$

To obtain a stable solution of the eigenvalue problem in Eq. (16), the kernel matrix $K_{cc}$ must be nonsingular. When $K_{cc}$ is singular, we can adopt the idea of regularization by adding a small constant value ρ to the diagonal of $K_{cc}$ as $K_{cc}+\rho I$ for any ρ>0. The matrix $K_{cc}+\rho I$ is nonsingular, and the projection vectors can be computed as the generalized eigenvectors of

(17) $$\left(K_{ct}H_{t}K_{tc}-\eta K_{cb}H_{b}K_{bc}\right)\text{A}=\Lambda \left(K_{cc}+\rho I\right)\text{A}.$$

### Animal model

Wild-type mice (strain #009104; n=12,9S4) were purchased from The Jackson Laboratory and housed in colony cages under pathogen-free conditions at The University of Texas MD Anderson Cancer Center Research Animal Support Facility. The mice were housed at an ambient temperature of 20–26°C and humidity range of 30–70% with a 12-h light-dark cycle. All animal experiments were conducted following MD Anderson Institutional Animal Care and Use Committee–approved protocols (approval number 00001217-RN03). For carcinogen-induced mouse models, a urethane-induced mouse model was used. Specifically, the 12,9S4 wild-type mice described above received intraperitoneal injections of 1 mg/g (body weight) urethane three times over 8 days when they were 6 weeks old. The mice were killed 7, 14, 20, 30, and 40 weeks after urethane administration, with a 0-week time point for mice that received no treatment. Both normal lung and lung tumor tissue samples were collected from the mice for downstream analysis.

### Single-cell sequencing and analysis

Fresh normal lung and lung tumor tissue samples collected from mice were immediately cut into pieces and placed in RPMI 1640 medium (Thermo Fisher Scientific) with 10% fetal bovine serum (FBS; Gibco). The tissue samples were enzymatically digested using a tumor dissociation mixture composed of 1 mg/ml collagenase A (Sigma), 0.4 mg/ml hyaluronidase (Sigma), and 1:5 bovine serum albumin fraction V (Thermo Fisher Scientific) according to the manufacturers’ instructions. Dissociation of tissue was carried out for 2 h on a rotary shaker at 37°C until all large tissue fragments were digested. Next, the dissociated tissues were transferred to conical tube and centrifuged at 350*g* for 5 min. The supernatant was removed, and 1–5 ml of prewarmed trypsin-EDTA was added to the collagenase/hyaluronidase-dissociated cells, resuspending them. Subsequently, 10 ml of cold RPMI 1640 without phenol red supplemented with 2% FBS was added and centrifuged at 350*g* for 5 min. As much of the supernatant as possible was collected, and 5 ml of prewarmed 5 U/ml dispase (STEMCELL Technologies) and 50 μl of DNase I solution (10 mg/ml in 0.15 M NaCl; STEMCELL Technologies) were added. The samples were pipetted for 1 min using a 1-ml micropipettor to further dissociate cell clumps. The cell suspension was diluted with an additional 10 ml of cold RPMI 1640 without phenol red supplemented with 2% FBS, and the cell suspension was filtered through a 40-μm Falcon cell strainer (Thermo Fisher Scientific) into a 50-ml tube. The cell suspension was further centrifuged at 450*g* for 5 min, and the supernatant was discarded. The pellet was resuspended in a 1:4 mixture of cold RPMI 1640 without phenol red supplemented with 2% FBS and an ammonium chloride solution (STEMCELL Technologies), which was followed by centrifugation at 450*g* for 5 min and discarding of the supernatant. Ten microliters of the cell suspension for each sample was analyzed using an automated cell counter (Thermo Fisher Scientific) to determine the number of live cells. Throughout the dissociation procedure, cells were kept on ice when possible. The cells were then loaded onto a Chromium single-cell controller (10x Genomics) to create single-cell gel beads in an emulsion according to the manufacturer’s protocol. ScRNA-seq libraries were constructed using a Single Cell 5′ Library and Gel Bead Kit v3.1 (10x Genomics) and sequenced using a NovaSeq 6000 sequencer (Illumina) at the Genomic and RNA Profiling Core at Baylor College of Medicine.

### Tissue preparation and ST

Normal and tumor tissue samples from mouse lungs were fixed in 10% formalin at room temperature for 24–48 h using a fixative volume 5–10 times greater than that of the tissue volume. Fixed tissues were transferred to 70% ethanol for temporary storage at 4°C. Paraffin embedding was conducted by the MD Anderson Research Histology Core Laboratory. Formalin-fixed, paraffin-embedded blocks were cut into 10-μm-thick sections using a precooled RNase-free microtome. These sections were then transferred onto Visium Spatial Gene Expression slides (10x Genomics), which were pretreated via floating in a water bath at 43°C. Following sectioning, the slides were dried at 42°C in a SimpliAmp Thermal Cycler (Thermo Fisher Scientific) for 3 h according to the manufacturer’s instructions. The slides were placed in a slide mailer, sealed with thermoplastic (Parafilm: Thermo Fisher Scientific), and stored overnight in a refrigerator at 4°C. The slides were then deparaffinized, fixed, stained with hematoxylin and eosin, and imaged at 5x magnification using a DM5500 B microscope (Leica Microsystems). Tile scans of the entire array were acquired using Leica Application Suite X software and merged. Spatial gene expression libraries (Visium ST; 10x Genomics) were processed according to the manufacturer’s instructions and sequenced using a NovaSeq 6000 sequencer (Illumina). All hematoxylin and eosin staining, imaging, library preparation, and sequencing processes were carried out at the Genomic and RNA Profiling Core at Baylor College of Medicine.

### Data processing

#### ScRNA-seq data.

Raw base call files were analyzed using Cell Ranger v.3.0.2 software (10x Genomics). The mkfastq command was used to generate FASTQ files, and the count command was used to generate raw gene-barcode matrices aligned to the GRCh38 Ensembl 93 genome. The data were aggregated using the cellranger aggr command, and further downstream analysis was conducted in R version 4.1.0 using the Seurat package (v.4.1.1). To ensure our analysis was performed using high-quality cells, filtering of cells was conducted by retaining cells that had unique feature counts greater than 200 or less than 5000 and had mitochondrial content less than 15%. After removing doublets, the total cell number was 70,698.

#### ST data.

The ST data sets were processed using Space Ranger software (v.2.0.1; 10x Genomics). The spatial sequencing data were aligned to mouse pre-mRNA genome reference version mm10 (downloaded from the 10x Genomics website) using Space Ranger, and mRNA count matrices were generated by adding intronic and exonic reads for each gene in each location. Paired histological hematoxylin and eosin stained images of tissues were processed using Space Ranger to select locations covered by tissue by aligning prerecorded spot locations with fiducial border spots in the images.

### Data analysis

#### ScRNA-seq analysis.

The scRNA-seq data were first normalized, and the 2000 most highly variable genes in the data were identified using variance-stabilizing transformation implemented in the Seurat package. Data were then scaled, and the first 30 principal components were extracted. The principal components were further transformed into the UMAP embedding space for which clustering analysis was conducted. The original Louvain algorithm was used for modularity optimization. The resulting 14 clusters were visualized in a 2D UMAP representation and annotated to known biological cell types using canonical marker genes. The following cell types were annotated (selected markers are listed in parentheses): endothelial cells (*Pecam1, Vwf, Ets1, Ace, Eng, Cldn5, and Mcam*), epithelial cells (*Epcam, Muc1, Cdh1, Krt7, and Krt8*), fibroblasts (*Pdpn, Dcn, Col3a1, Mgp, Col1a1, and Col6a1*), macrophages (*Apoe, C1qa, C1qb, C1qc, Marco, Mrc1, Fabp4, Inhba, Ccl4, Cxcl10, Rsad2, and Herc6*), conventional dendritic cells (*cDC; H2-Aa, Ccr7, Flt3, Fscn1, and Clec9a*), proliferating macrophages (*Mki67, Tubb5, and Tuba1b*), B cells (*Cd19, Ms4a1, Cd79a, Cd79b, and Blnk*), T cells (*Trbc2, Cd2, Cd3d, Cd3e, Cd3g, Cd4, Cd8a, Cd8b1, Il2ra, and Foxp3*), proliferating T cells (*Mki67, Tubb5, and Tuba1b*), plasmacytoid dendritic cells (*pDC; Siglech, Ly6c2, and Cd209d*), neutrophils (*S100a8, S100a9, and Csf3r*), plasma cells (*Sdc1, Mzb1, Xbp1, and Jchain*), monocytes (*Cd14, Fcgr4, Lst1, and Vcan*), and natural killer cells (*Nkg7, Klrg1, and Ncr1*).

#### ST analysis.

The raw expression count matrices for both the background and target ST data sets were normalized using variance-stabilizing transformation implemented in the Seurat package. The normalized data were then standardized to have zero mean and unit standard deviation. The standardized expression data matrices with 3000 genes were then used as inputs to our CoCo-ST method for low-dimensional feature representation. Clustering on the UMAP-embedded learned contrastive feature representations was then performed. Further differential gene expression analysis was conducted, and spatial domains were annotated based on the differentially expressed marker genes.

### Pathway analysis

The most important genes (the 20 genes with the largest weights) on the top five contrastive components were identified, and the biological processes associated with these contrastive components were examined. Specifically, gene set enrichment analysis was performed with these 20 genes with the largest weights in the loading matrix using the g:GOSt function in the gprofiler2 package. In this analysis, all of the input 3000 genes were used as the background, and the default options in the g:SCS method in gprofiler2 were used for multiple testing correction. The gene sets were downloaded from the Molecular Signatures Database, including the KEGG, Gene Ontology biological processes, Gene Ontology cellular components, and Gene Ontology molecular functions.

### Cell type deconvolution

Cell type deconvolution in ST enables estimation of cell type composition on each spatial location by leveraging a reference scRNA-seq data set. Cell type deconvolution was performed using the RCTD^23^ method implemented in the spacexr R package. ScRNA-seq data for the same mouse lung tumor samples (MLP samples) served as the reference data for deconvolution. The reference data contained 70,698 cells of multiple immune and malignant types as described in the scRNA-seq analysis section. The RCTD method was run in doublet mode to estimate the reference cell type composition on each spatial location. Other parameters were set to the default settings.

### Cell-cell interaction

Cell-cell interaction for the ST data sets was performed using CellChat^24^. The CellChatDB.mouse database of ligand-receptor interactions specifically curated for mice was used to identify overexpressed ligand-receptor interactions. The group-level communication probability or interaction weights were then computed using the truncated mean method with a 10% truncated mean. Subsequently, the communication probability at the signaling pathway level was computed by summarizing the communication probabilities of all ligand-receptor interactions associated with each signaling pathway. Finally, the cell-cell communication network was aggregated by summarizing the overall communication probabilities.

### Trajectory inference analysis

For spatial trajectory analysis of individual tissue samples, the low-dimensional contrastive feature representations were used as inputs to the Slingshot algorithm^25^. Slingshot was applied to the contrastive feature representations so that nearby tissue spatial locations with similar gene expression would have similar pseudotimes. Because Slingshot requires predefined cluster labels, the spatial domain labels from the spatial domain identification analysis were used for Slingshot. The normal lung spatial domain was set as the start cluster (beginning of the trajectory or pseudotime) with a focus on trajectory inference on tumor and tumor-adjacent spatial domains to determine how these locations are connected to one another during tumorigenesis.

For the trajectory analysis with combined tissue samples, spots belonging to normal lung, adenoma, and adenocarcinoma spatial domains as determined using the contrastive feature representations were collected, and Monocle3^26^ was used to infer the trajectory. First, the combined data (spots)were processed using the standard Seurat approach, including total count normalization, scaling, and PCA analysis. Next, UMAP embedding was determined, which was used to learn the trajectory that fits the spots’ UMAP coordinates. A principal graph was then fit on the UMAP embedding, and the spots were ordered according to their progress along the learned trajectory. To identify genes that varies among spot clusters in the UMAP embedding space, spatial autocorrelation analysis (Moran’s I) was performed, and the obtained variable genes were grouped into modules by determining UMAP embedding of the genes followed by gene clustering based on Louvain community detection analysis.

## Supplementary Material

1

## Figures and Tables

**Fig. 1 |:**
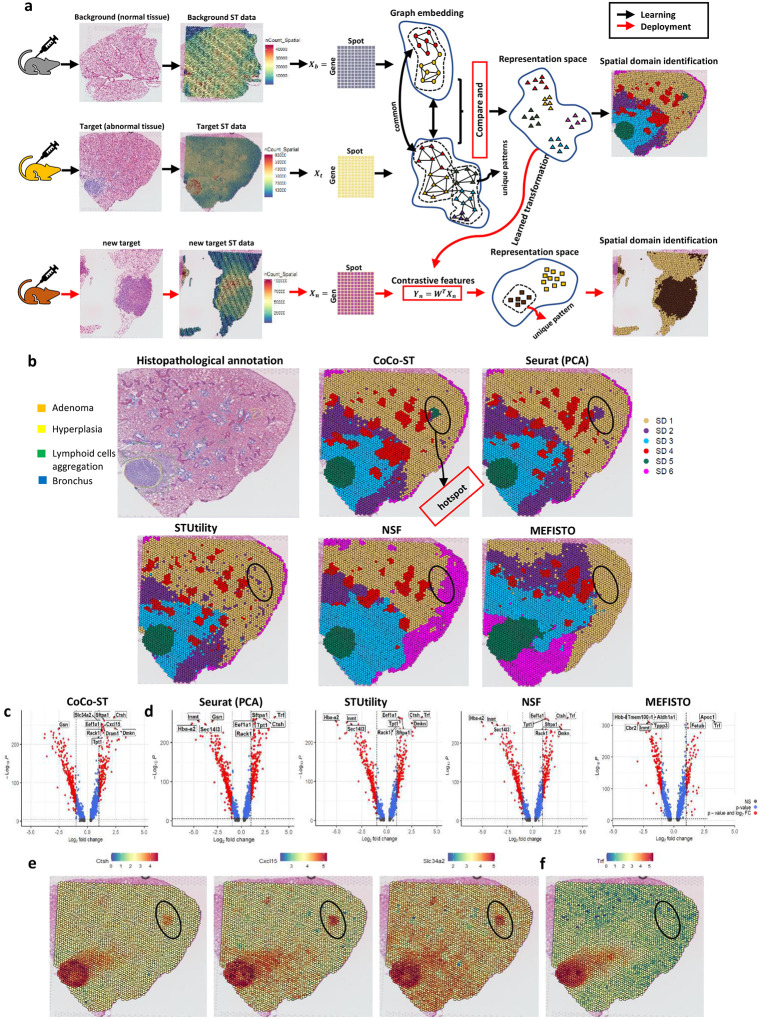
CoCo-ST identifies unique, interesting spatial structures enriched in ST data sets. **a**, Overview of the CoCo-ST workflow. **b**, A target ST tissue sample containing unique, interesting spatial structures annotated by a pathologist and spatial domains/regions identified using the different feature representation methods. **c**, Volcano plot of the most differentially expressed genes for the adenoma spatial domain identified by CoCo-ST. **d**, Volcano plot of the most differentially expressed genes for the adenoma spatial domain identified using the compared approaches. **e**, Spatial expression patterns for the most differentially expressed genes (Ctsh, Cxcl15, and Slc34a2) for the adenoma spatial domain identified using CoCo-ST. These genes had high expression patterns in both the larger and smaller (hotspot) adenoma spatial domains. **f**, Spatial expression pattern for the most differentially expressed gene (Trf) for the adenoma spatial domain identified using the compared approaches. This gene had high expression pattern only within the larger adenoma spatial domain, with no such pattern observed in the smaller (hotspot) region.

**Fig. 2 |:**
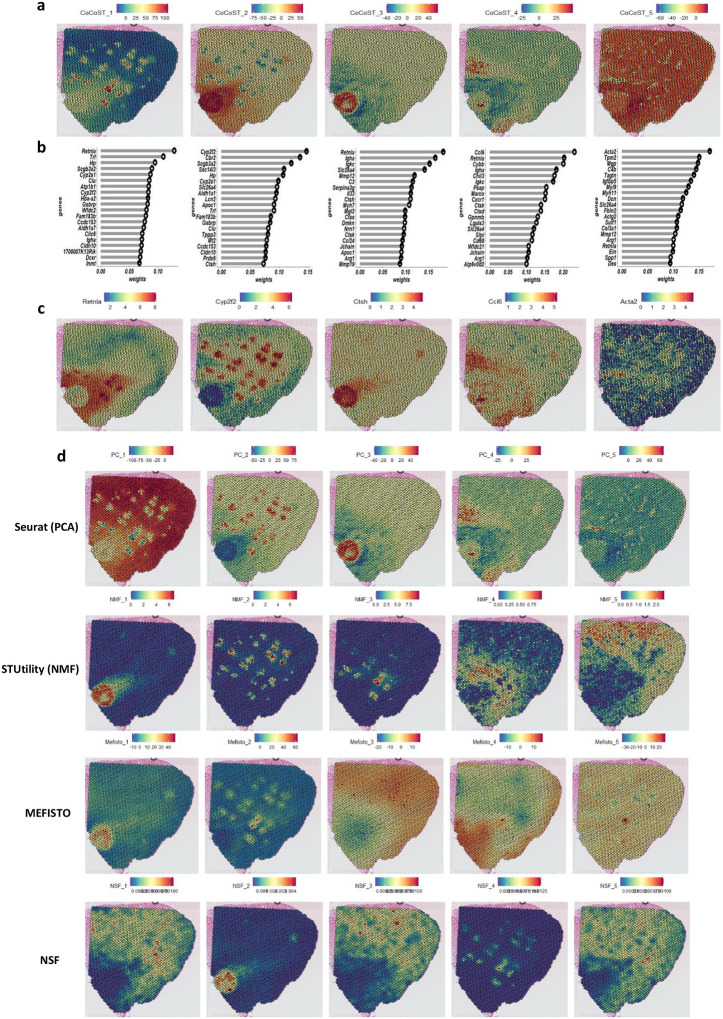
CoCo-ST’s contrastive components marked interesting spatial structures enriched in ST data sets. **a,** Spatial patterns captured by the first five contrastive components of CoCo-ST. **b**, The top 20 genes with the largest weights on the corresponding first five contrastive components. Symbols to the right of the bars indicate the signs of the weights. **c**, Expression patterns for the top representative genes for each of the first five contrastive components. **d**, Spatial patterns captured by the first five components of the compared approaches.

## Data Availability

The scRNA-seq and ST data sets analyzed in this study will be made available upon reasonable request through a data access agreement with the corresponding authors.
