# Supplementary material for: CoCo-ST: Comparing and Contrasting Spatial Transcriptomics data sets using graph contrastive learning
Source: Res Sq. 2024 May 20:rs.3.rs-4359834. Preprint. [Version 1] doi: 10.21203/rs.3.rs-4359834/v1 (PMC11142361; doi:10.21203/rs.3.rs-4359834/v1)
Supplement: 1 [file NIHPPRS4359834V1-supplement-1.pdf]

Supplementary Information

Extended Data Fig. 1 | Spatial domains identified on all MLP tissue samples using CoCo-ST's contrastive components. The similarity graphs for both the background and target ST data sets were constructed based on the molecular data sets.

Extended Data Fig. 2 | Differential gene expression analysis of detected spatial domains. a, UMAP embedding of the contrastive components determined using CoCo-ST on the target ST tissue sample. **B**, UMAP embedding of spotsshowing the expression of some of the most differentially expressed genes in different clusters identified using the contrastive feature representations from CoCo-ST. **c**, Violin plots of the expression levels for the most differentially expressed genes for the different spatial domains identified using CoCo-ST. **d**, Biological processes and pathways associated with the 10 most differentially expressed genes for the adenoma spatial domain detected using CoCo-ST. **e**, Violin plots of the expression levels for the most differentially expressed genes for the different spatial domains identified using the compared feature representation approaches.

Extended Data Fig. 3 | Biological processes and pathways associated with CoCo-ST's contrastive components.

Extended Data Fig. 4 | Spatial domains identified on all MLP tissue samples using CoCo-ST's contrastive components. The similarity graphs for both the background and target ST data sets were constructed based on spatial locations.

Extended Data Fig. 5 | Application of CoCo-ST's contrastive components to studying cell-cell interaction at different cancer stages. a, UMAP embedding of the scRNA-seq data set used as a reference for cell type deconvolution. **b**, Spatial domains identified in the MLP-6 tissue sample using CoCo-ST's contrastive components. **c**, Cell type annotation on each of the spatial locations in MLP-6 tissue sample as inferred by the RCTD deconvolution algorithm. **d**, Percentage of different cell types (y-axis) in the different spatial domains (x-axis) detected using CoCo-ST. **e**, Cell-cell interaction weight plot for MLP-6 tissue sample. The thicker the line, the stronger the interaction between the cell types. **f**, Chord plot of the cell-cell interactions via canonical WNT signaling. **g**, Heat map of the communication probabilities for WNT signaling from senders (sources) to receivers (targets). **h**, Heat map of network centrality scores for WNT signaling highlighting the major signaling roles of the different cell groups.

Extended Data Fig. 6 | Predicted spatial distributions of major cell types in the MLP-6 tissue sample.

Extended Data Fig. 7 | Distribution of different cell types in each spatial domain on the MLP-6 tissue sample determined using CoCo-ST. The cell type percentages in each spatial domain add up to 100%.

Extended Data Fig. 8 | Aggregated cell-cell interaction plots on the combined MLP tissue samples containing the adenoma and adenocarcinoma spatial domains. a, Cell-cell interaction weight plot for the adenocarcinoma-related MLP tissue samples. **b**, Simplified cell-cell interaction plots for **a** showing signaling sent from each cell group. The thicker the line, the

stronger the communication. **c**, Cell-cell interaction weight plot for the adenoma-related MLP tissue samples. **d**, Simplified cell-cell interaction plots for **c** showing signaling sent from each cell group. The thicker the line, the stronger the communication.

Extended Data Fig. 9 | Application of CoCo-ST's contrastive components to trajectory inference (cancer evolution). a, Spatial trajectory inference based on CoCo-ST's determined contrastive components. The arrows indicate the direction of the trajectory, which points from the normal lung spatial domain to the adenoma spatial domain. **b**, Learned trajectory pseudotime, with red- to green-colored regions indicating tissue locations with low and high pseudotime. **c**, UMAP embedding of spots belonging to the combined normal, adenoma, and adenocarcinoma spatial domains as determined using CoCo-ST. **d**, Trajectory inference of the cancer evolution from normal tissue to adenoma to adenocarcinoma colored according to their corresponding pseudotimes. **e**, Heat map of gene modules containing differentially co-expressed genes that vary across the different stages of cancer as determined from the learned trajectory in **d**. **f**, Bar plot of the number of differentially co-expressed genes in each module in **e**.

Extended Data Fig. 10 | Application of CoCo-ST to a mouse brain ST data set. a, Spatial domains/regions identified on anterior and posterior mouse brain tissue samples based on CoCo-ST's contrastive components. **b**, Spatial patterns on the anterior mouse brain tissue sample captured by the first five contrastive components of CoCo-ST. **c**, The 20 genes with the largest weights on the first five contrastive components in **b**. Symbols to the right of the bars indicate the signs of the weights. **d**, Expression patterns for some representative genes in **c**. **e**, Spatial patterns on the posterior mouse brain tissue sample captured by the first five contrastive

components of CoCo-ST. **f**, The 20 genes with the largest weights on the first five contrastive components in **e**. **g**, Expression patterns for some representative genes in **f**.

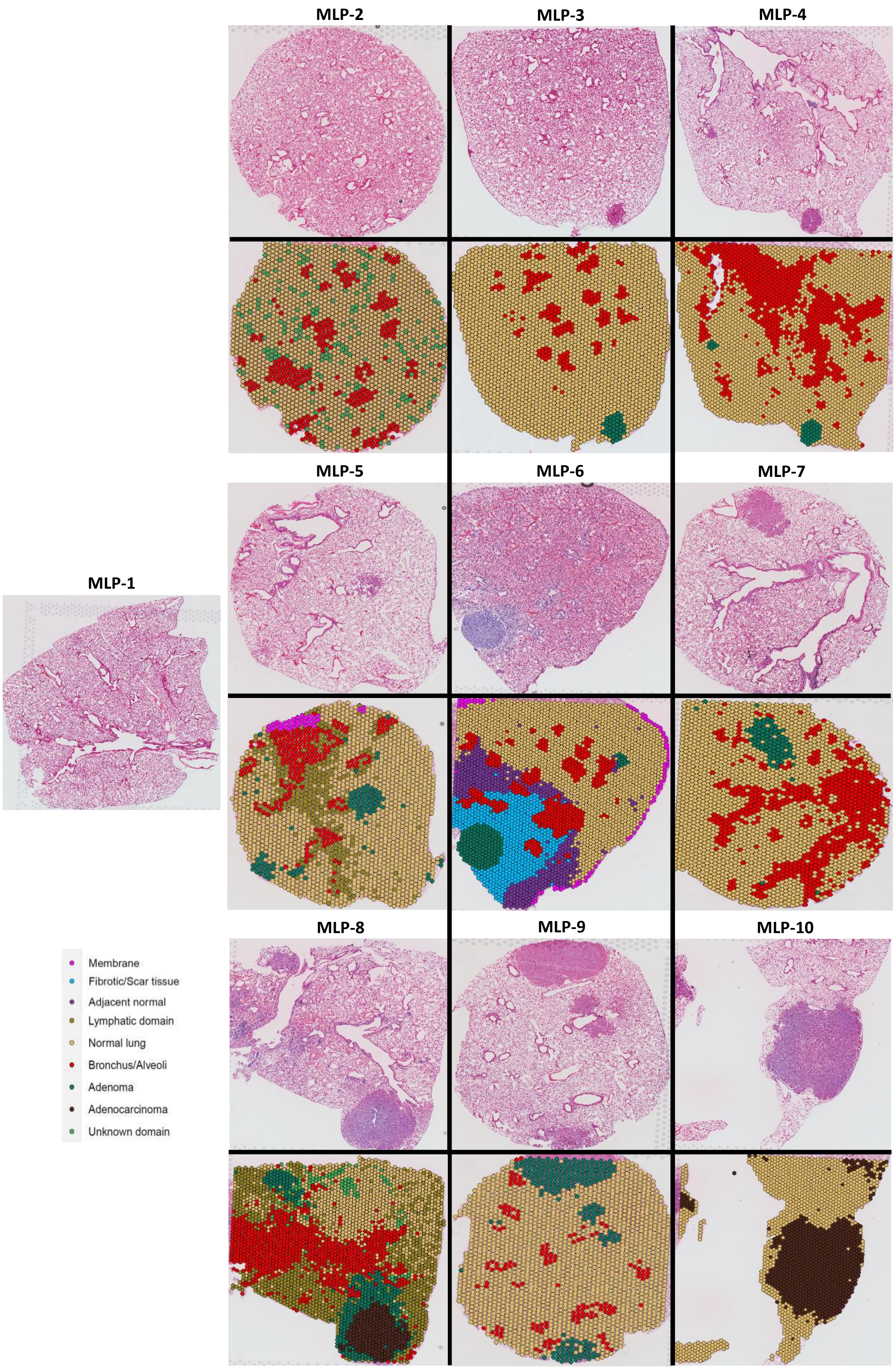

Extended Data Fig. 1

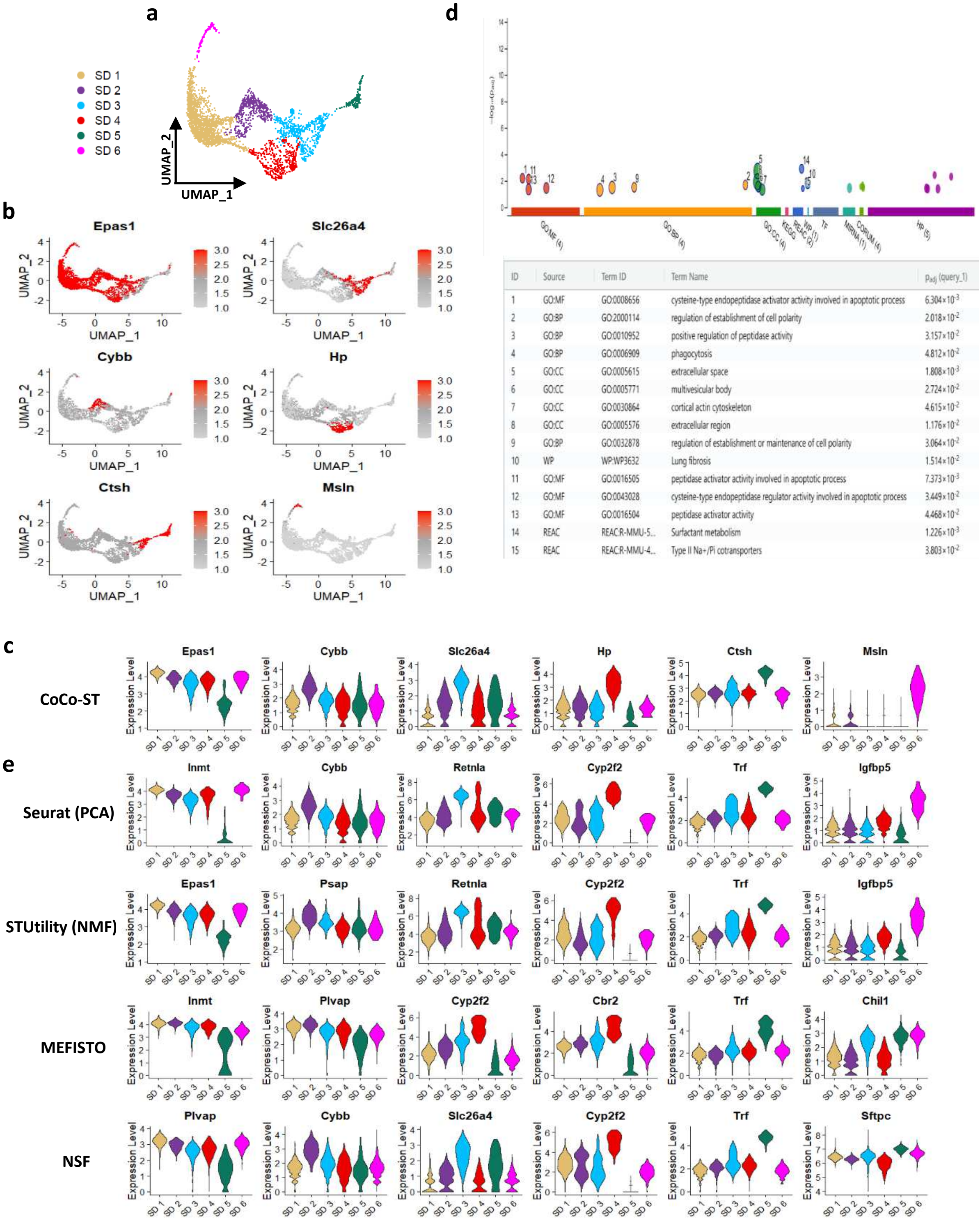

ID	Source	Term ID	Term Name	p_{adj} (query_1)
1	GO:MF	GO:0008656	cysteine-type endopeptidase activator activity involved in apoptotic process	6.304×10^{-3}
2	GO:BP	GO:2000114	regulation of establishment of cell polarity	2.018×10^{-2}
3	GO:BP	GO:0010952	positive regulation of peptidase activity	3.157×10^{-2}
4	GO:BP	GO:0006909	phagocytosis	4.812×10^{-2}
5	GO:CC	GO:0005615	extracellular space	1.808×10^{-3}
6	GO:CC	GO:0005771	multivesicular body	2.724×10^{-2}
7	GO:CC	GO:0030864	cortical actin cytoskeleton	4.615×10^{-2}
8	GO:CC	GO:0005576	extracellular region	1.176×10^{-2}
9	GO:BP	GO:0032878	regulation of establishment or maintenance of cell polarity	3.064×10^{-2}
10	WP	WP:WP3632	Lung fibrosis	1.514×10^{-2}
11	GO:MF	GO:0016505	peptidase activator activity involved in apoptotic process	7.373×10^{-3}
12	GO:MF	GO:0043028	cysteine-type endopeptidase regulator activity involved in apoptotic process	3.449×10^{-2}
13	GO:MF	GO:0016504	peptidase activator activity	4.468×10^{-2}
14	REAC	REAC:R-MMU-5...	Surfactant metabolism	1.226×10^{-3}
15	REAC	REAC:R-MMU-4...	Type II Na+/Pi cotransporters	3.803×10^{-2}

Extended Data Fig. 2

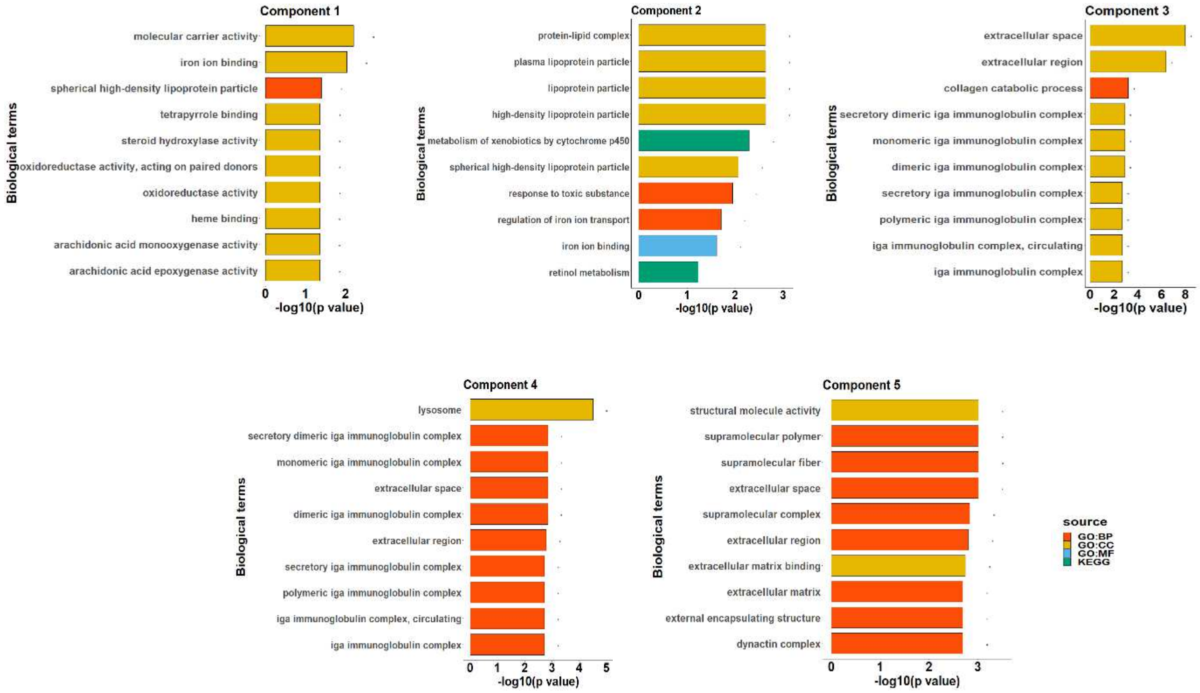

Extended Data Fig. 3

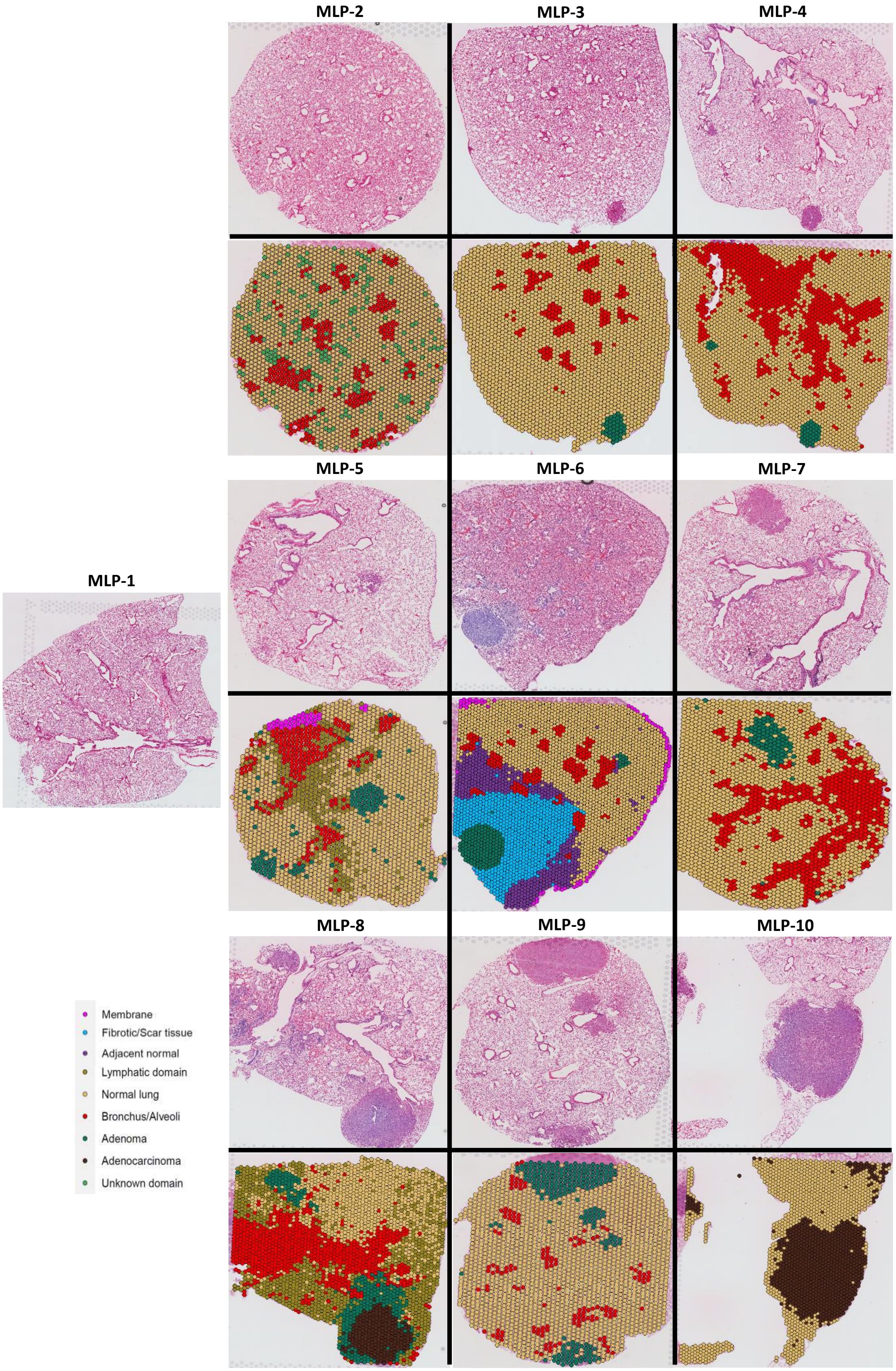

Extended Data Fig. 4

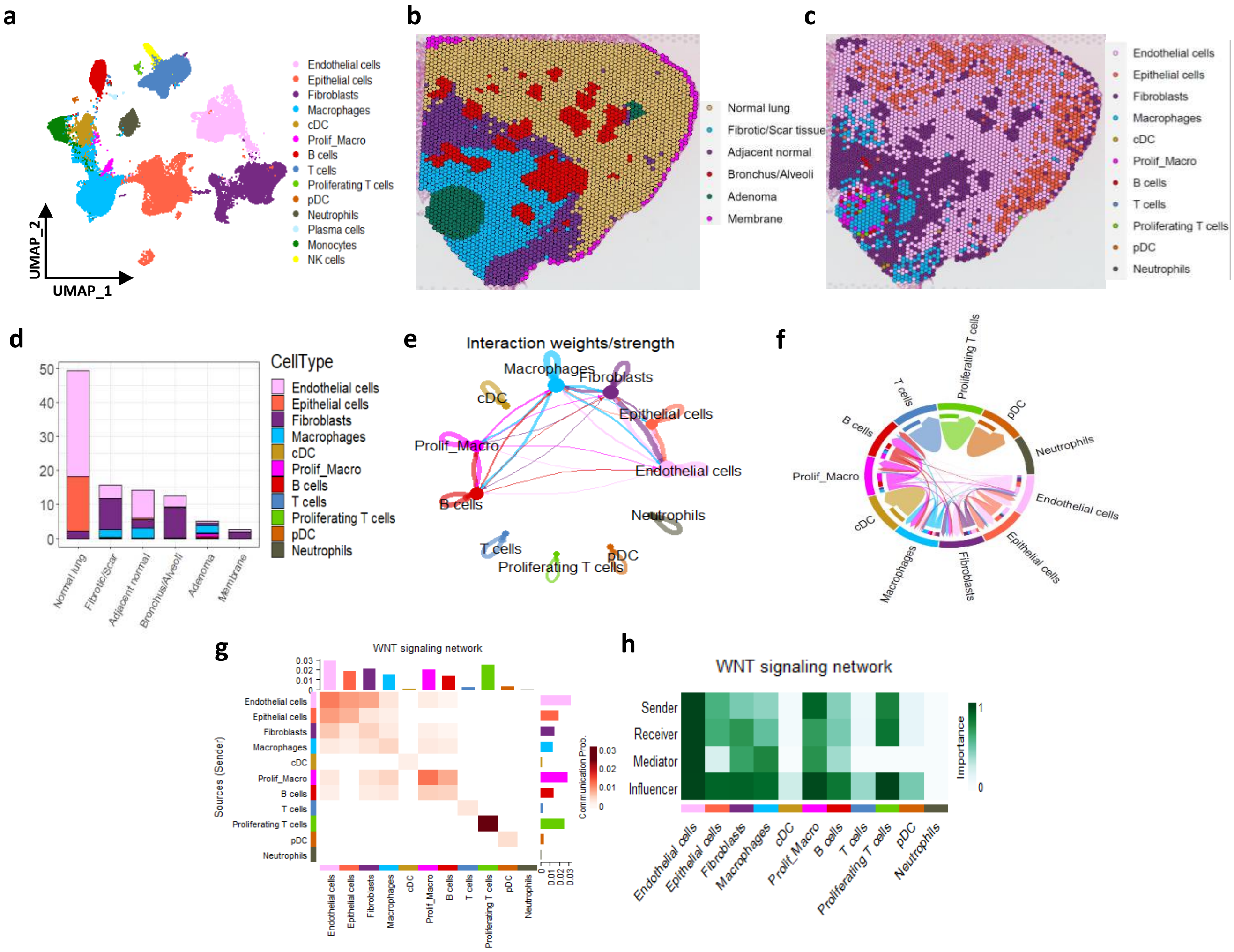

Extended Data Fig. 5

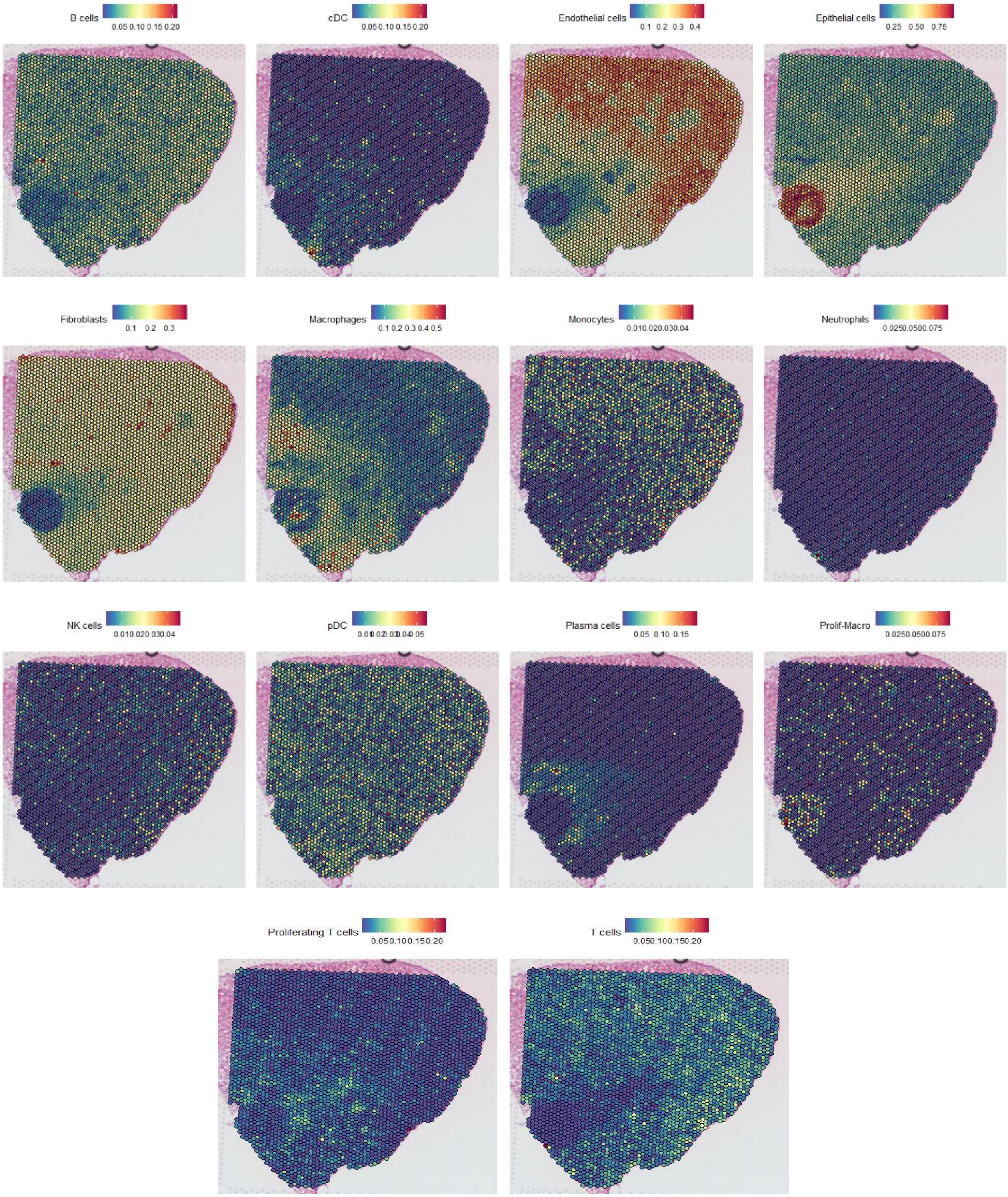

Extended Data Fig. 6

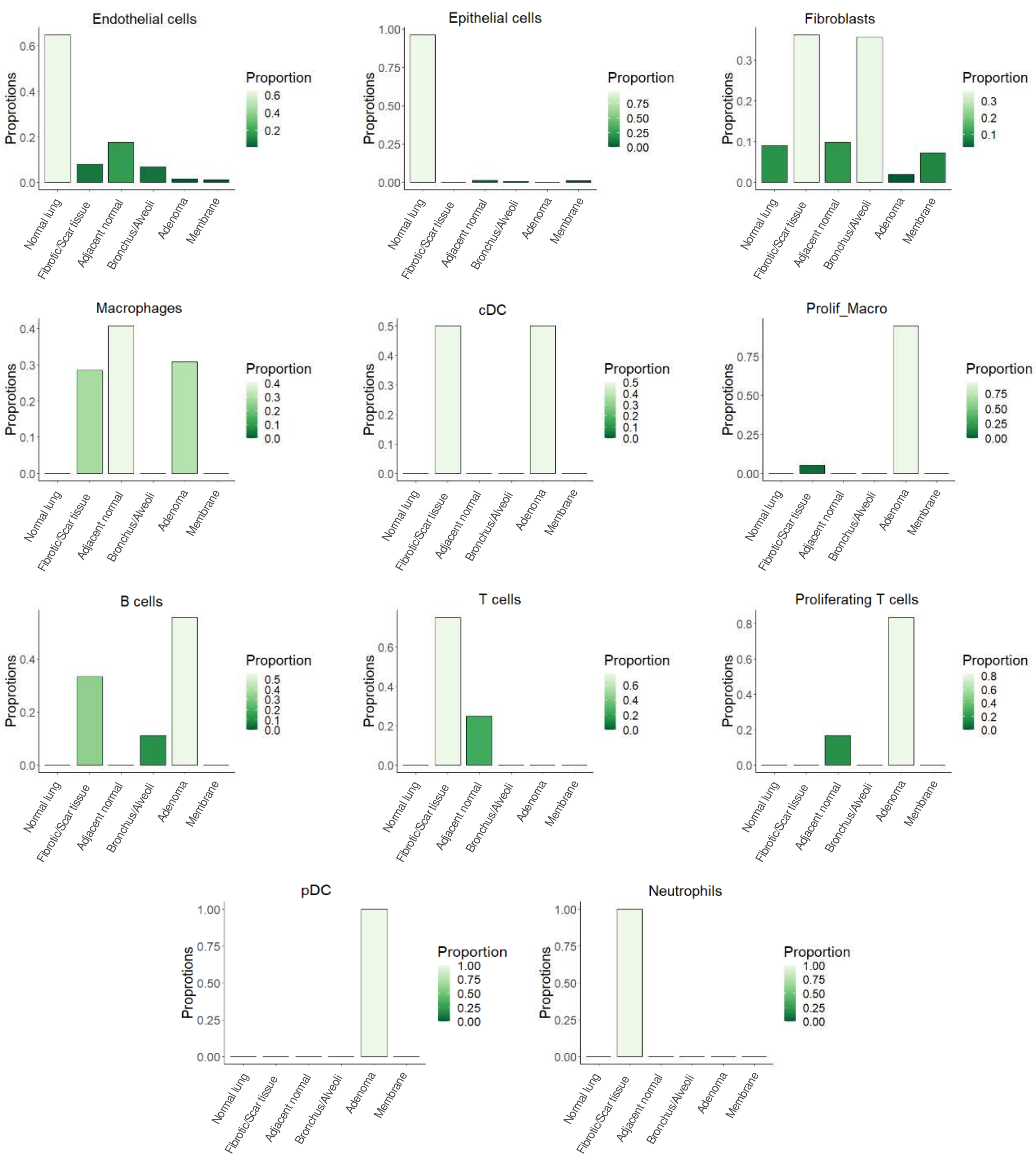

Extended Data Fig. 7

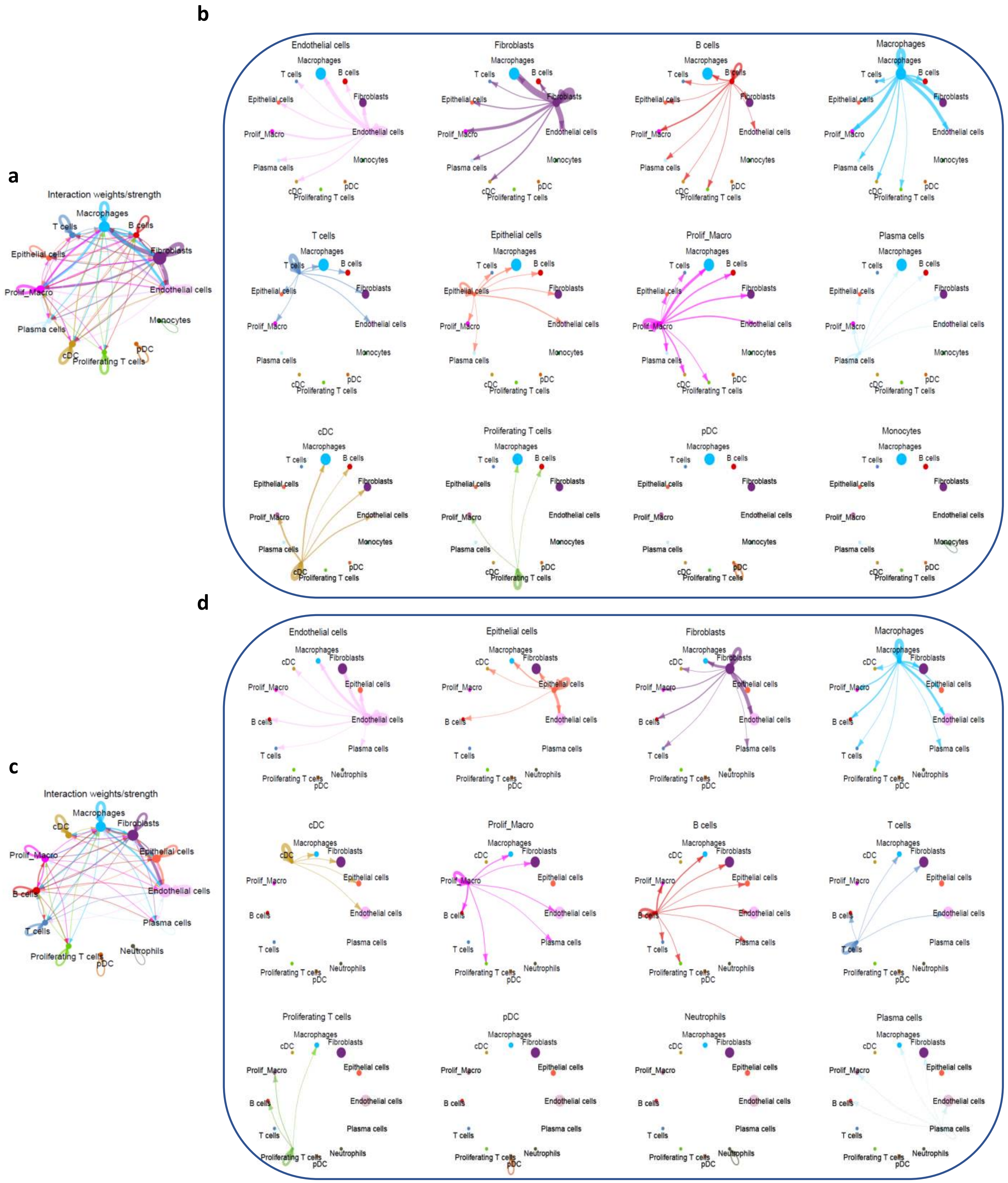

Extended Data Fig. 8

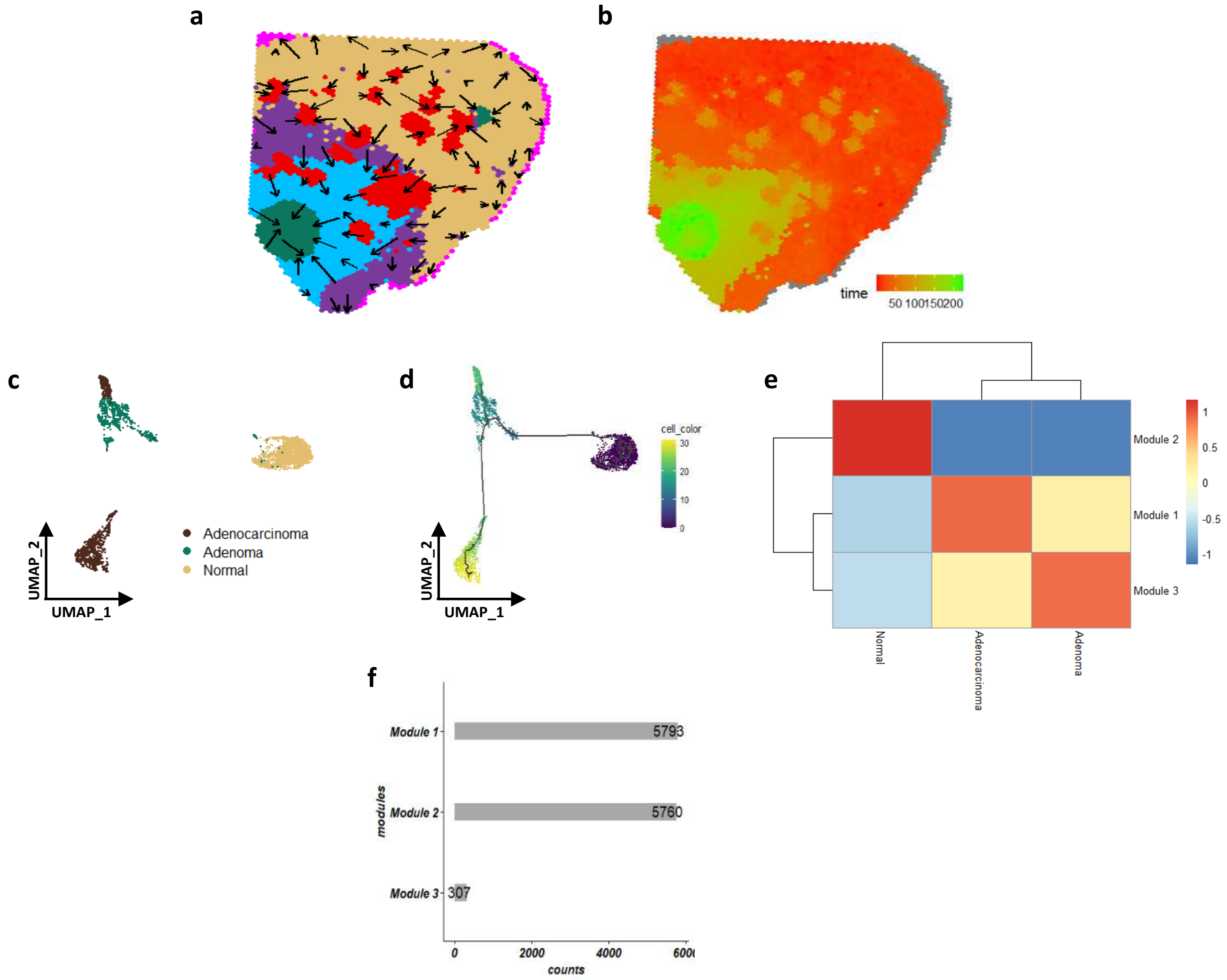

Extended Data Fig. 9

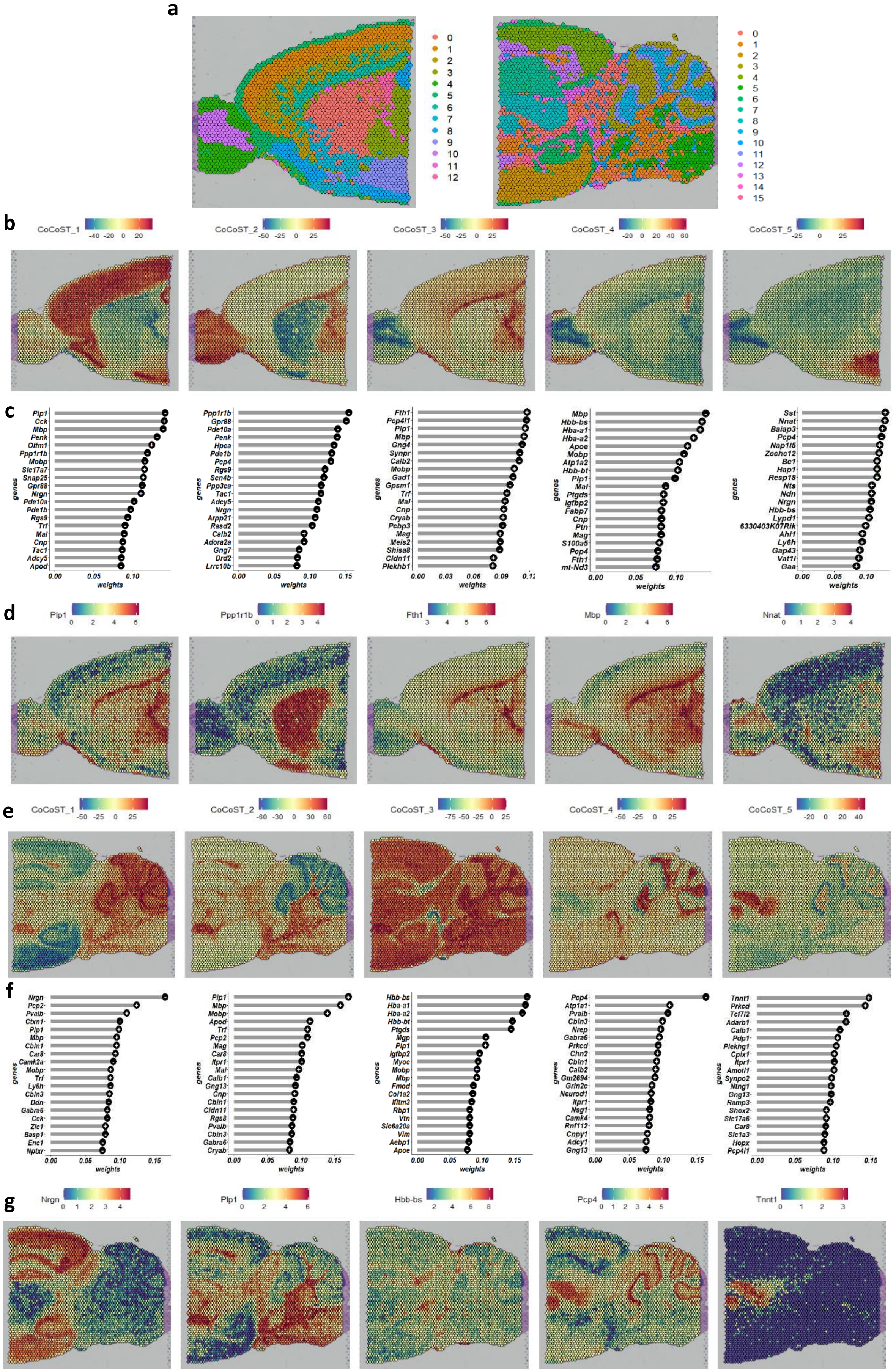

Extended Data Fig. 10
